# Imaging patterns and recommendations for diagnosis, staging, and management of lung cancer

**DOI:** 10.1093/bjro/tzaf013

**Published:** 2025-05-21

**Authors:** Nivedita Chakrabarty, Abhishek Mahajan, Nitin Shetty, Naveen Mummudi, Devyani Niyogi, Falguni Hota, Deepak Dabkara, Reefath Jebraj, Nilendu Purandare, Vanita Noronha, Ashu Bhalla, Kumar Prabhash

**Affiliations:** Department of Radiodiagnosis, Tata Memorial Centre, Advanced Centre for Treatment, Research and Education in Cancer (ACTREC), Homi Bhabha National Institute (HBNI), Parel, Mumbai, Maharashtra 400 012, India; Department of Imaging, The Clatterbridge Cancer Centre NHS Foundation Trust, L7 8YA Liverpool, UK; Faculty of Health and Life Sciences, University of Liverpool, L7 8TX, Liverpool, UK; Department of Radiodiagnosis and Interventional Radiology, Tata Memorial Centre, Advanced Centre for Treatment, Research and Education in Cancer (ACTREC), Homi Bhabha National Institute (HBNI), Parel, Mumbai, Maharashtra 400 012, India; Department of Radiation Oncology, Tata Memorial Centre, Tata memorial Hospital, Homi Bhabha National Institute (HBNI), Parel, Mumbai, Maharashtra 400 012, India; Department of Surgical Oncology, Tata Memorial Centre, Tata memorial Hospital, Homi Bhabha National Institute (HBNI), Parel, Mumbai, Maharashtra 400 012, India; Department of Interventional Radiology, Tata Memorial Centre, Tata memorial Hospital, Homi Bhabha National Institute (HBNI), Parel, Mumbai, Maharashtra 400 012, India; Department of Medical Oncology, Tata Medical Center, Rajarhat, Kolkata 700 160, West Bengal, India; Department of Radiodiagnosis, Tata Memorial Hospital, Tata Memorial Centre, Homi Bhabha National Institute (HBNI), Parel, Mumbai, Maharashtra 400 012, India; Department of Nuclear Medicine and Molecular Imaging, Tata Memorial Centre, Tata Memorial Hospital, Homi Bhabha National Institute (HBNI), Parel, Mumbai, Maharashtra 400 012, India; Department of Medical Oncology, Tata Memorial Centre, Tata Memorial Hospital, Homi Bhabha National Institute (HBNI), Parel, Mumbai 400 012, Maharashtra, India; Department of Radiology, All India Institute of Medical Sciences (AIIMS), New Delhi 110029, India; Department of Medical Oncology, Tata Memorial Centre, Tata Memorial Hospital, Homi Bhabha National Institute (HBNI), Parel, Mumbai 400 012, Maharashtra, India

**Keywords:** lung cancer, imaging patterns, imaging guidelines, NCCN, ESMO, ASCO, interventional radiology

## Abstract

Lung cancer is the second most commonly diagnosed cancer worldwide. In the present era of targeted therapy for various lung cancer mutations, it is essential to be aware of the imaging correlates of various lung cancer mutations on contrast enhanced computed tomography of thorax. In this article, we have discussed the imaging patterns of various types of lung cancer including different mutations and also comprehensively reviewed the imaging recommendations (National Comprehensive Cancer Network [NCCN], European Society of Medical Oncology [ESMO] and American Society of Clinical Oncology [ASCO]) and management guidelines of lung cancer (non-small cell, small cell and other neuroendocrine tumours). We have also discussed guidelines for screening, diagnosis, staging (recent 9th edition tumour node metastasis [TNM]), treatment response evaluation, and follow up. Role of interventional radiology in the treatment of primary lung cancer, lung metastasis, and management of posttreatment complications, have also been described in detail in this article. In addition, current status of artificial intelligence in lung cancer has also been briefly discussed.

## Introduction

As per the Global Cancer Observatory (GLOBOCAN) 2020 data, lung cancer is the second most commonly diagnosed cancer worldwide with 2.2 million new cases in 2020 and 1.8 million (18%) cancer-related deaths worldwide.^1^^,^^2^ Of all the lung cancer types, non-small-cell lung cancer (NSCLC), which includes adenocarcinoma and squamous cell carcinoma, comprises 84%, small cell lung cancer (SCLC) comprises 13%, and other lung neuroendocrine tumour comprises 1%–2%.^1^^,^^3^^,^^4^ Pulmonary NeuroEndocrine Tumours (NETs) are classified by the World Health Organisation into: (1) Low grade: Typical carcinoids (TC), (2) Intermediate grade: Atypical carcinoids (AC), and (3) High grade: Large cell neuroendocrine carcinoma (LCNEC) and small cell lung carcinoma (SCLC).^5^

Knowledge of imaging patterns of different types of lung cancer, including common molecular mutations, as well as those of brain metastases originating from lung cancer, can aid in treatment planning. Through this article, we intend to update the readers (radiologists and clinicians) regarding the current imaging guidelines for lung cancer screening, diagnosis, staging, treatment response evaluation, and follow up. Principles of management of initial as well as recurrent lung cancers along with the role of interventional radiology (IR) in treatment of primary lung cancer, lung metastasis, and management of posttreatment complications, have also been discussed in detail. Overview on the current status of artificial intelligence in lung cancer, has also been briefly discussed.

## Epidemiology and clinical presentation

Lung cancer tops the list of cancer-related deaths worldwide.^2^ Cigarette smoking is strongly associated with an elevated risk of lung cancer, with the risk growing higher as the duration and intensity of smoking increases.^6^^,^^7^ SCLC and squamous cell carcinoma have a higher association with smoking compared to LCNEC and adenocarcinoma.^8^ Passive smoking also enhances the risk of lung cancer.^6^^,^^7^ Non-cigarette tobacco products such as cigars, pipes, smokeless tobacco, electronic cigarettes, and occupational lung carcinogens such as asbestos, diesel fumes, arsenic, beryllium, cadmium, chromium, nickel, and silica, have positive association with lung cancer.^7^^,^^9^ Among non-smokers, radon is most common cause of lung cancer.^7^ There is increased risk of lung cancer with air pollution and particulate matter (PM_2.5_).^7^ Non-smoking women are at increased risk of having lung cancer compared to non-smoking men. Also, a higher proportion of women have epidermal growth factor receptor (EGFR) mutations in NSCLC and an increased incidence of adenocarcinoma with lepidic features.

Women with resected NSCLC show frequent KRAS mutation as compared to men with resected NSCLC.^10^ Cough, haemoptysis, hoarseness of voice (due to tumour/node involving aortopulmonary window) and weight loss are usual presenting symptoms of lung cancer.^11^ Those presenting with metastasis usually have symptoms pertaining to the organ of involvement, for example, headache, confusion in case of brain metastasis, bone and back pain in case of bone metastasis, right upper quadrant pain, anorexia in case of liver metastasis.^11^ SCLC can also present with superior vena cava obstruction or due to mediastinal invasion or right paratracheal lymphadenopathy.^11^ In addition, patients can also present with paraneoplastic syndrome, which is more common in SCLC compared to NSCLC.^1^^,^^12^

## Clinical and laboratory diagnostic work-up

Smoking history, comorbidities, and performance status should be evaluated for all the patients having lung cancer. Once the tissue diagnosis of NSCLC is established, histological subtype through immunohistochemistry (IHC), molecular testing, and radiological stage needs to be determined.^13^ Molecular profiling should be performed for all the classic actionable biomarkers, for example, EGFR, anaplastic lymphoma kinase (ALK), BRAF, ERBB2 (HER2), KRAS, METex14 skipping, Neurotrophic tyrosine receptor kinase (NTRK) 1/2/3, rearranged during transfection (RET), and ROS1.^14^ Following are the prevalence of actionable mutations among NSCLC patients: ALK: 4.1%-21.4%, BRAF: 1.5%-3.5%, EGFR: 11.9%-51.8%, HER2: 0%-1.5%, KRAS: 4.5%-6.4%, NTRK: 0%-.7%, and ROS-1: 3.5%-4.1%.^15^

Essential blood investigations for SCLC include complete blood count, renal and liver function tests, serum calcium & lactate dehydrogenase (LDH).^16^ Pulmonary function test is required before starting treatment in localized small cell carcinoma.^16^ In SCLC, molecular profiling should be done only in extensive stage SCLC for patients who have never smoked tobacco.^11^

## Imaging referral guidelines

National Comprehensive Cancer Network (NCCN) and European Society of Medical Oncology (ESMO) recommend contrast enhanced computed tomography (CECT) thorax with upper abdomen for the characterization of primary tumour (T) and regional nodes (N) in NSCLC, and fluorodeoxyglucose positron emission tomography CECT (FDG PET-CECT) for detection of extracerebral metastasis.^14^^,^^17^ Contrast enhanced magnetic resonance imaging (CEMRI) brain should be used in stages II, III, and IV of NSCLC for screening of brain metastasis as per the NCCN, ESMO, and American Society of Clinical Oncology (ASCO) recommendations.^1^^,^^17^ MRI brain for metastasis screening can also be done for large central tumour (>5 cm) in stage IB of NSCLC as per the NCCN.^1^ CECT brain is recommended if CEMRI brain is not available for metastasis screening.^1^ In SCLC, in addition to CECT thorax, CECT abdomen and pelvis and CEMRI brain should be performed for all the stages as per the NCCN recommendations.^11^ NCCN, ESMO, and *ASCO* have proposed posttreatment surveillance guidelines on imaging which have been described in the section on “follow up.”^11^^,^^14^^,^^16–18^

### Screening

5-year overall survival (OS) of stage IA lung cancer ranges from 70% to 84.2%; however, more than 75% individuals are detected at an advanced incurable stage.^19^^,^^20^ The National Lung Screening Trial (NLST) study showed a reduction in lung cancer mortality with 3 rounds of annual low dose CT (LDCT) screening, compared to chest radiographs, for high-risk smokers aged 55-74 years. The Nederlands-Leuvens Longkanker Screenings Onderzoek (NELSON) study also found a reduction in lung cancer mortality with 4 rounds of LDCT screening at increasing intervals, compared to no screening, for high-risk smokers aged 50-74 years.^21^ National Lung Screening Trial recommends screening using LDCT to reduce mortality from lung cancer.^19^ As per the NCCN guidelines, patients who belong to high risk category, that is, age ≥50 years and ≥20 pack-year history of smoking cigarettes, should undergo screening with LDCT.^14^ Various international body recommendations for lung cancer screening are shown in Table 1.^22^ LDCT is the modality of choice for screening of lung cancer and Lung CT Screening Reporting & Data System (Lung-RADS) should be followed for reporting and taking decisions for further management and follow up based on findings on LDCT.^14^^,^^23^

**Table 1.** Lung cancer screening recommendations by various international bodies.

International body	Recommendations
American Association of Thoracic Surgery	Annual low-dose CT scan screening for high-risk individuals (ages 55 to 79 years with ≥30 pack-year history of smoking and current smoker or quit within past 15 years; ages 50 to 79 years with ≥20 pack-year history and cumulative risk >5% over next 5 years; or lung cancer survivors with no incidence of disease for ≥4 years).
US Preventive Services Task Force	Screening annual low-dose CT scan screening for high-risk individuals (ages 50 to 80 years with a 20 pack-year history of smoking and current smoker or quit within past 15 years). Discontinue when person has not smoked for 15 years or if limited life expectancy.
American College of Chest Physicians	Annual low-dose CT scan screening for high-risk individuals (ages 55 to 77 years with ≥30 pack-year history of smoking and current smoker or quit within past 15 years).
American Society of Clinical Oncology	Annual low-dose CT scan screening for high-risk individuals (ages 55 to 74 years with ≥30 pack-year history of smoking and current smoker or quit within past 15 years).

### Diagnostic imaging, staging, and interventions

The ninth edition TNM staging (TNM-9); developed by the International Association for the Study of Lung Cancer (IASLC) and adopted by the American Joint Committee on Cancer (AJCC) and the Union for International Cancer Control (UICC), has come into effect from January 1, 2025.^24–27^ In TNM-9, changes have been made in the N2 and M1 subcategories based on the differences in prognostic and survival outcomes, and availability of new treatment options.

N2 category has been divided into N2a (single-station involvement) and N2b (multiple-station involvement), owing to better prognostic implications and survival outcomes of N1a as compared to N2b subcategories.^25^^,^^26^^,^^28^^,^^29^ This necessitates that radiologists should use the regional lymph node stations according to the IASLC lymph node map and mention all the involved nodal stations instead of number of nodes.^24^^,^^25^^,^^30^ Certain categories have been downstaged by this new TNM stage, expanding the options for curative intent treatment.^24^^,^^25^  Table 2 enlists the changes in 9th edition TNM stage classification due to division of N category.^28^^,^^29^

**Table 2.** Changes in the 9th edition Node (N) category affecting the Tumur Node Metastasis (TNM) stage classification of lung cancer.

T and N categories	Previous (8th edition) stage classification	Current (9th edition) stage classification
T1 N1	IIB	IIA
T1 N2a	N2a and N2b categories were non-existent. T1N2 was classified as IIIA	IIB
T1 N2b		IIIA
T2 N2a	N2a and N2b categories were non-existent. T2 N2 was classified as IIIA	IIIA
T2 N2b		IIIB
T3 N2a	N2a and N2b categories were non-existent. T3 N2 was classified as IIIB	IIIA
T3 N2b		IIIB
T4 N2a	N2a and N2b categories were non-existent. T4 N2 was classified as IIIB	IIIB
T4 N2b		IIIB

M1c (multiple metastases) has been subcategorized into M1c1 (multiple metastases in a single organ system) and M1c2 (multiple metastases in multiple organ systems).^24^^,^^25^^,^^27^ A single organ system could mean a single organ, paired organs (for example lungs), or an organ system present throughout the body (for example, skin, skeletal system). As per TNM-9, number of metastases in a single organ has no bearing on the stage.^24^ Multiple metastases; irrespective of subcategories, are classified as stage IVB.

#### Role of CECT

CECT thorax with upper abdomen including adrenals for NSCLC, and CECT thorax, abdomen and pelvis for SCLC, is the modality of choice for detection and characterization of primary tumour (T); location, size, and relation of the tumour to the adjacent structures, for evaluation of regional nodes (N), and for ruling out metastases to the contralateral lung, liver and adrenal glands.^14^^,^^17^ For part-solid tumours, the size of the solid component on CT, which corresponds to the invasive component, defines the T category based on tumour size.^31^ In most of the cases, radiologists can raise the suspicion of lung cancer on CECT and suggest biopsy for confirmation. If nodes are not suspicious on CECT and the tumour is in the peripheral third of lung with size <3 cm, then pathologic confirmation is optional as the likelihood of positive mediastinal lymph nodes is low in such cases, otherwise all other nodes require pathologic confirmation.^14^ As per the NCCN recommendation, if there is a strong clinical suspicion of stage IA lung cancer (based on risk factors and radiologic appearance), then biopsy is not required for confirmation.^14^ CT scan is excellent for detection of rib and vertebral erosion as well as for vascular invasion as well. In addition, the condition of the underlying lung (interstitial lung disease, chronic obstructive pulmonary disease) can also be well depicted on CECT thorax which has implication on management.^32^ The dose of contrast administered for a typical protocol of CECT thorax is 1-1.5 ml/kg with a iodine concentration of 300-350 mgI/ml.^33^ CT is the modality of choice for performing image guided biopsy of lung tumours.

##### Imaging patterns of various types of lung cancer

*Adenocarcinoma.* Adenocarcinoma in situ typically presents as a ground glass density nodule ≤3 cm in size suggestive of pure lepidic growth, without any tumour necrosis. However, they can also show part-solid content or internal bubble-like lucencies.^34^ Minimally invasive adenocarcinoma presents as ≤3 cm sized ground glass nodule with central ≤5 mm solid component. The invasive component is usually solid and the non-invasive component is usually ground glass on CT scan.^34^ Lepidic predominant adenocarcinoma is typically >3 cm sized part-solid nodule or mass on CT scan.^34^ Invasive mucinous adenocarcinomas can be unifocal or multicentric lesions with lower lobe predominance, presenting as consolidation with air bronchograms or as solid and subsolid nodules and masses with a bronchogenic distribution.^34^ Studies have shown that patients with EGFR mutations in lung adenocarcinoma usually have peripherally located well-defined ground glass opacities (GGO), or mixed GGO, air bronchogram, and pleural retraction. Whereas, ALK mutation usually present as solid tumours, absence of air bronchogram and metastasis.^35^^,^^36^

*Squamous cell carcinoma*. Squamous cell carcinoma frequently shows cavitation due to tumour necrosis. Squamous cell carcinoma can also grow peripherally around a central scar.^37^

*Pulmonary neuro endocrine tumours.* SCLC presents as a centrally located mass inseparable from hilar or mediastinal lymphadenopathy.^38^ Typical carcinoid presents as a well-defined, spherical or ovoid endobronchial mass showing intense enhancement.^38^ LCNEC does not have any typical imaging feature.^37^^,^^38^

**Figure 1.**
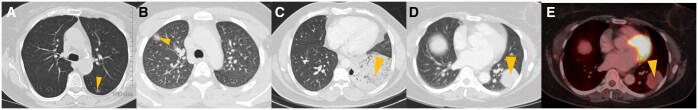
(A-E) Various patterns of lung adenocarcinomas on contrast enhanced computed tomography (CECT) thorax. (A) CECT thorax shows a pure ground glass nodule (arrowhead) in this biopsy proven adenocarcinoma in situ. (B) CECT thorax shows a ground glass nodule with a small (≤5 mm) central solid component (arrowhead) in this biopsy proven minimally invasive adenocarcinoma. (C) CECT thorax shows central bubble-like lucencies (arrowhead) within left lower lobe mass, in this biopsy proven lepidic predominent adenocarcinoma. (D, E) CECT thorax (D) shows left lower lobe solid mass (arrowhead) without any fluorodeoxyglucose uptake on positron emission tomography CT (E), in this biopsy proven mucinous adenocarcinoma.

**Figure 2.**
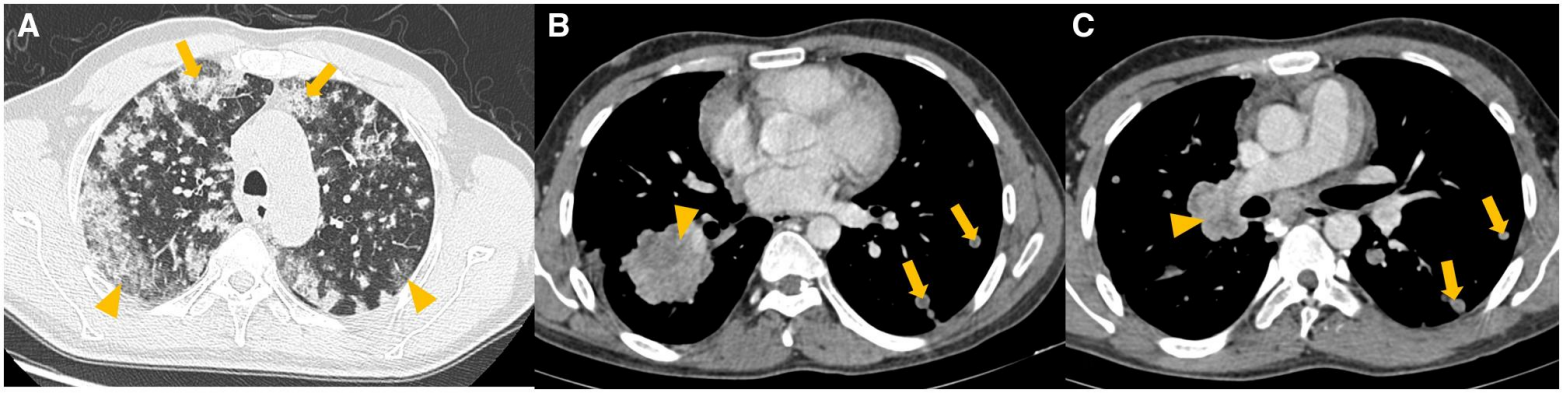
(A-C) Contrast enhanced computed tomography (CECT) lung with epidermal growth factor receptor (EGFR) and anaplastic lymphoma kinase (ALK) mutation positive lung adenocarcinomas. (A) CECT thorax shows multifocal discrete and confluent nodules; some pure ground glass (arrowheads) and some part-solid (arrows), in this 60 years old female (without any history of smoking) with biopsy proven EGFR mutation positive lung adenocarcinoma. (B, C) CECT thorax (mediastinal window) of a 50 years old male (without any history of smoking) with biopsy proven ALK mutation positive lung adenocarcinoma, shows a solid right lower lobe mass with irregular margins (arrowhead in B) and metastatic left lung nodules (arrows in B, C). In addition, metastatic right hilar nodal mass (10R station) is seen (arrowhead in C).

**Figure 3.**
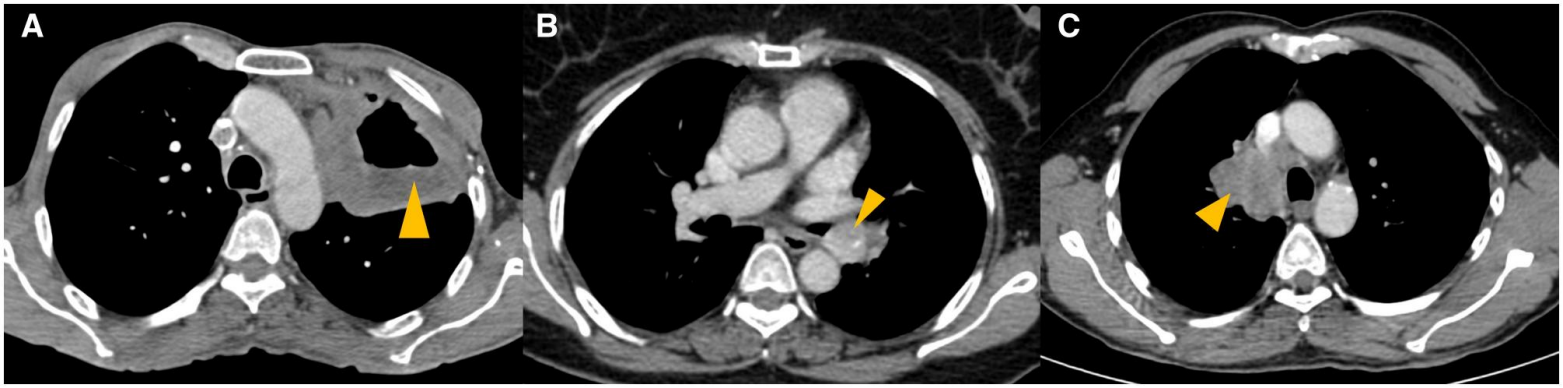
(A-C) Contrast enhanced computed tomography (CECT) of types of lung cancer. (A) CECT thorax (mediastinal window) shows cavitation within a solid left upper lobe lung mass (arrowhead), in this 70 years old male (with history of smoking) with biopsy proven squamous cell carcinoma. (B) CECT thorax (mediastinal window) of a 45 years old male with biopsy proven typical carcinoid, shows an enhancing endobronchial lesion in left main bronchus (arrowhead). (C) CECT thorax (mediastinal window) in this 75 years old male (with history of smoking) with biopsy proven small cell lung cancer, shows centrally located, right suprahilar mass conglomerating with, and inseparable from right paratracheal nodes (arrowhead).

**Figure 4.**
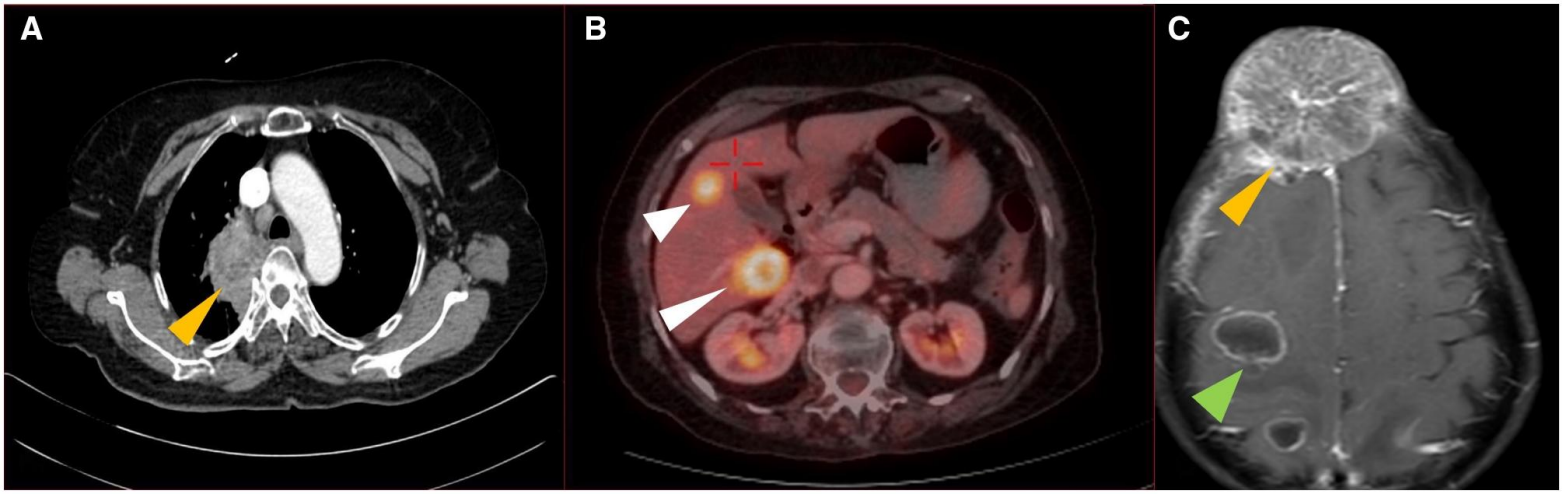
62 years old male with stage IVB squamous cell carcinoma. (A) Right perihilar mass (arrowhead) infiltrating the mediastinum with metastatic level 2R node. (B) Liver metastases (arrowheads) showing avidity on fluorodeoxyglucose positron emission tomography computed tomography (FDG PET/CECT). (C) MRI brain showing brain parenchymal metastasis in right precentral gyrus (green arrowhead) and frontal bone calvarial metastasis (yellow arrowhead).

Imaging patterns of various types of lung carcinomas are shown in Figures 1-4.

#### Role of PET-CECT

FDG PET-CECT performed from skull base to knees or whole body is recommended for lung cancer staging in order to detect extracerebral metastasis (Figure 4).^14^ FDG PET-CECT may be omitted in a resource constraint setting, if CECT already shows liver/adrenal metastasis in NSCLC. Pathologic or other radiologic confirmation is warranted if there is a positive PET-CECT scan for distant disease, and pathologic confirmation of lymph node is required for PET-CECT positive status in the mediastinum.^14^ However, in case of <3 cm peripheral tumours (outer third), pathologic confirmation is optional as there is a low likelihood of positive mediastinal lymph nodes in such cases.^14^ FDG PET-CECT can also guide biopsy planning as the site showing maximum avidity within the tumour can be targeted for biopsy. Invasive mucinous adenocarcinoma has variable FDG uptake.^39^

A typical FDG PET-CECT protocol entails 0.1 mCi/kg of 2-[18F]-fluoro-2-deoxy-d-glucose intravenous injection 45 to 90 min before the examination and CT is performed by giving 125 ml (1-2 ml/kg) of iodinated contrast medium at the rate of 4 ml/s using a power injector.^1^ For suspected TC, the choice of imaging is a DOTANOC PET-CECT.^40^

#### Role of MRI

CEMRI is the modality of choice for brain metastasis screening (Figure 4), both parenchymal as well as leptomeningeal.^1^^,^^17^^,^^41^ A typical protocol for brain metastasis is shown in Table 3.^1^ European Association for Neuro-Oncology (EANO) and ESMO have jointly proposed recommendations for cerebrospinal MRI, consisting of CEMRI of the brain and post-contrast sagittal T1-weighted sequence of the spinal axis, in those suspected of having leptomeningeal metastasis.^41^ CE T1W imaging is the most important sequence for diagnosing leptomeningeal disease.^42^ MRI can accurately assign T category for Pancoast tumours by providing information on chest wall infiltration (T3), involvement of brachial plexus (T4), subclavian vessels (T4) and vertebral body (T4), which can help decide resectability or alternative management. In addition, the status of spinal cord can be best evaluated by MRI for lung tumours that show neural foraminal and intraspinal extension. Operable lung cancer with equivocal pericardial involvement on CECT may warrant MRI for better evaluation.^32^

**Table 3.** Contrast enhanced magnetic resonance imaging (CEMRI) protocol for brain metastasis.

Parameters	Description
Sequences	2D T2WI, 2D DWI, SWI/T2*weighted GRE, pre and post contrast 3D T1WI (FSPGR or FSE), 2D FLAIR post contrast
Slice thickness for 3 T MRI	1 mm for post contrast and 3 mm for rest of the sequences
Slice thickness for 1.5 T MRI	≤1.5 mm for post contrast and ≤4 mm for rest of the sequences
Imaging plane	Sagittal or axial plane for post contrast sequence and axial plane for rest of the sequences
Contrast dose	0.1 mmol/kg using gadolinium chelated contrast agent @ 3–5 cc/sec

2D T2WI: two-dimensional T2-weighted imaging, 2D DWI: two-dimensional diffusion-weighted imaging, SWI/GRE: susceptibility-weighted imaging/gradient echo sequences, FSPGR: fast spoiled gradient echo, FSE: fast spin echo, FLAIR: fluid-attenuated inversion recovery.

##### Imaging patterns of brain metastasis

Brain metastasis can be solid or cystic and usually located at the gray-white matter junction or in the watershed territories.^1^ One of the studies have shown that brain metastasis from EGFR positive mutations have infiltrative borders on T2WI, whereas, EGFR negative metastasis have well-defined margins.^1^ EGFR wild group commonly showed focal restriction on DWI.^1^ Another study showed that brain metastasis with infiltrative margins and enhancing T2 hypointense peripheral solid rim with diffusion restriction, were usual features in ALK positive mutations.^1^ Whereas, well-defined margins with uniform or patchy enhancement, and central diffusion restriction were features of ALK negative mutations.^1^

Comparative performance of different imaging modalities for lung cancer staging are shown in Table 4.^43–54^

**Table 4.** Comparative performance of different imaging modalities for lung cancer staging.

Study	Imaging modality	T status	N status	Adrenal metastasis (Stage IV)	Brain metastasis (Stage IV)	Bone metastasis (Stage IV)
Wever et al T staging of NSCLC^43^	FDG PET/CECT	PPV = 86%	PPV = 75% NPV = 90% S = 83% Sp = 84% A = 84%			
	CECT	PPV = 68%	PPV = 60% NPV = 88% S = 83% Sp = 68% A = 74%			
Suh et al^44^	FDG PET/CECT		*For solid portion of tumour ≤1 cm* Sp = 87.5% NPV = 98.9% A = 86.6% *For solid portion of tumour >1-3 cm* S = 50% Sp = 74.8% PPV = 14.1% NPV = 94.8% A = 72.9%			
	CECT		*For solid portion of tumour ≤1 cm* Sp = 97% NPV = 99% A = 96% *For solid portion of tumour > 1-3 cm* S = 13.6% Sp = 98.1% PPV = 37.5% NPV = 93.2% A = 91.7%			
Sung et al^45^	FDG PET			S = 74% Sp = 73% A = 74%		
	FDG PET/CT			S = 80% Sp = 89% A = 84%		
Park et al^46^	FDG PET/CT			*For lesion with high FDG uptake compared to liver* S = 82.4% Sp = 92.2% PPV = 77.8% NPV = 94%		
	Adrenal protocol CT			S = 88.2% Sp = 84.3% PPV = 65.2% NPV = 95.6%		
Kruger et al^47^	FDG PET/CT				S = 27.3% Sp = 97.6% PPV = 75% NPV = 83.3%	
Ferrigno et al^48^	CECT				S = 92% Sp = 99% A = 98%	
Rodrigues et al^49^	FDG PET/CT					S = 97.7% Sp = 100% A = 99.4%
	Bone scintigraphy					S = 87.8% Sp = 97.5% A = 94.2%
Zidan et al^50^	PET-CT				S = 78.13% Sp = 92.65% A = 88%	
	CECT				S = 81.25% Sp = 94.12% A = 90%	
Kitajima et al^51^	PET				S = 45% Sp = 80% A = 66%	
	CT				S = 50% Sp = 97% A = 78%	
	PET-CT				S = 50% Sp = 93% A = 76%	
Li et al^52^	PET CT				S = 21%	
	MRI				S = 77%	
Schoenmaekers et al^53^	CECT				Detected brain metastasis in 7% of stage III NSCLC	
	CEMRI				Additional 4.7% brain metastases detected in the same stage III NSCLC.	
Khosla et al^54^	CECT				S = 92% Sp = 99% A = 98%	

CECT: Contrast enhanced computed tomography FDG PET: Fluorodeoxyglucose positron emission tomography S= Sensitivity, Sp= Specificity, A= Accuracy, PPV = Positive Predictive Value, NPV = Negative Predictive Value.

#### Role of diagnostic interventions

##### For primary tumour/metastasis

CT guided percutaneous needle biopsy (PNB) is the procedure of choice to diagnose lung tumour and has a diagnostic yield of around 90%, but for nodules smaller than 1.5 cm, the yield drops to 70% to 80%.^55–58^ Complications occur in about 25% of patients undergoing CT-guided PNB; pneumothorax in 15% to 25% of patients requiring chest tube placement in 7%, and significant haemorrhage in about 1% of patients.^56^^,^^59^ As per the Cardiovascular and Interventional Radiological Society of Europe (CIRSE) guideline, lung lesion biopsy is of moderate risk category.^60^ Indications and contraindications of biopsy are mentioned in Table 5.^61^

**Table 5.** Indications and contraindications of biopsy.

Indications	To establish the benign or malignant nature of a suspected tumour To classify a malignancy (including immunohistochemistry [IHC] evaluation) To stage a patient with known (or suspected) malignant tumours elsewhere To obtain material for molecular analysis
Absolute contraindications	Lack of a safe access; Non-correctable coagulopathy; Refusal by the patient
Relative contraindications	Coagulopathies (biopsy can be performed after correction) Inability of patient to cooperate (general anaesthesia may be considered); Significant comorbidities (ie, haemodynamic or respiratory instability); Pregnancy (since ionizing radiation exposure is required).

##### For nodes

There are various methods for mediastinal staging/tissue diagnosis, for example, mediastinoscopy, mediastinotomy, endobronchial ultrasound (EBUS), endoscopic ultrasound (EUS), navigational bronchoscopy, robotic bronchoscopy and CT-guided biopsy and the least invasive biopsy with the highest yield is preferred for initial diagnosis.^14^

##### Image localization and access route

After evaluating the limited CT images needle trajectory can be planned in multiplanar reformatted images. Basically the needle path should be as short as possible and should avoid all risky structures like lung fissures and bullae, large vessels. In special situations, a longer route is recommended for easy manipulation, for example, in subpleural lesions, a longer oblique intraparenchymal needle is recommended.^62^

For suspected TC, EBUS guided biopsy is the procedure of choice for diagnosis.^11^

EUS can be used to guide biopsy from left adrenal gland. Patients having pleural effusion with lung cancer should undergo thoracentesis and cytology.^11^^,^^14^ Solitary metastasis needs biopsy confirmation and in case of multiple metastases, confirmation from one site is required, if possible.^14^ If biopsy from the metastatic site is risky or technically not feasible in a suspected case of lung carcinoma with multiple metastases, biopsy can be performed from the primary tumour or mediastinal nodes.^14^

Follow up and audit should be done for each and every patient.

##### Role of navigational bronchoscopy (NB)

It offers a safer, minimally invasive alternative to CT-guided PNB, with fewer complications. Pneumothorax occurs in just 1.6% of patients, chest tube placement in 0.7%, and haemorrhage in 0.5%. An additional benefit of NB is its ability to simultaneously sample multiple lung nodules and mediastinal or hilar lymph nodes in one procedure. However, its diagnostic yield is generally lower than CT-PNB, ranging from 38% to 47% in registry studies and up to 73% in the largest prospective study to date.^56^^,^^63^ Digital tomosynthesis enables correction of CT scan-body divergence through intraprocedure 3-D visualization of isolated pulmonary nodules using standard 2-D fluoroscopy. This technology has recently been integrated into the SuperDimension NB platform by Medtronic. Recent studies have shown diagnostic yields of around 80%, which are comparable to the yields reported in the CT-guided transthoracic needle biopsy (CT-PNB) literature.^56^^,^^64^^,^^65^

### Response assessment imaging in neoadjuvant, adjuvant, and palliative setting

Post chemotherapy response of primary tumour and extracerebral metastasis is evaluated using the latest version of Response Evaluation Criteria in Solid Tumors (RECIST 1.1) on CECT.^66^ Post immunotherapy, response is assessed using immune RECIST (iRECIST) that entails a repeat confirmation CECT scan 4 to 8 weeks later to account for the phenomenon of pseudoprogression, which has 5% incidence in advanced NSCLC.^67^

Response assessment in neuro-oncology brain metastases (RANO BM) and RANO immunotherapy (iRANO) are used for evaluating response of brain metastasis after chemotherapy and immunotherapy respectively.^60^ Positron Emission Tomography Response Criteria in Solid Tumors (PERCIST) based on metabolic response on FDG PET/CECT, is not routinely used for response evaluation unless this criteria is a part of a clinical trial.^68^

### Follow-up

NCCN, ASCO and ESMO have laid down imaging surveillance guidelines after definitive therapy (curative intent) for lung cancer (both NSCLC and SCLC), which have been described in Table S1.^11^^,^^14^^,^^16–18^ None of these guidelines recommend FDG PET/CECT or MRI brain for surveillance in NSCLC.^11^^,^^14^^,^^16–18^

## Principles of management in lung cancer

### NSCLC

Patients with early-stage, node negative (Stage IA) NSCLC are usually managed with single modality; lobectomy or sub-lobar resection being the most common approaches with reported 5-year overall survival (OS) of 80%-85%.^69^ Patients deemed inoperable due to poor lung function or significant co-morbidities are treated using stereotactic ablative radiotherapy (SABR) with reported outcomes similar to surgery.^70^

Addition of 4-6 cycle of platinum doublet chemotherapy after surgical excision has been well-established in the management of stage II NSCLC with an approximate 5-year OS of 50%-60%.^71–73^ Adjuvant Osimertinib and Atezolizumab has also been incorporated into the recent guidelines in EGFR mutant and ≥1% Programmed Cell Death Ligand-1 (PDL1) expressing tumours respectively.^74–76^

Post-operative margin positive status is the only indication for adjuvant radiotherapy (RT) in post-operative setting.^77^

Locally advanced tumours with mediastinal nodal involvement (Stage III) are normally managed with combined modalities which includes 6-7 weeks of conventional RT with concurrent chemotherapy with 5-year OS of 20%-30%.^78^ Addition of adjuvant Durvalumab after definitive chemoradiotherapy has been shown to improve survival outcome significantly.^78^ Stage IV metastatic NSCLC is mostly managed with palliative systematic chemotherapies with palliative radiotherapy reserved for symptomatic sites with extremely poor long-term survival. First line targeted monotherapies (e.g. EGFR antagonists, ALK inhibitor, etc.) and immunotherapies (PD1/PDL1 inhibitors) have been well-established in tumours expressing targetable mutations, and has become the standard of care.^79^ De novo oligometastatic disease with limited 3-5 metastasis can be managed with ablative high dose radiotherapy to metastatic sites with local consolidative therapy to primary and has been shown to improve survival.^80^

**Figure 5.**
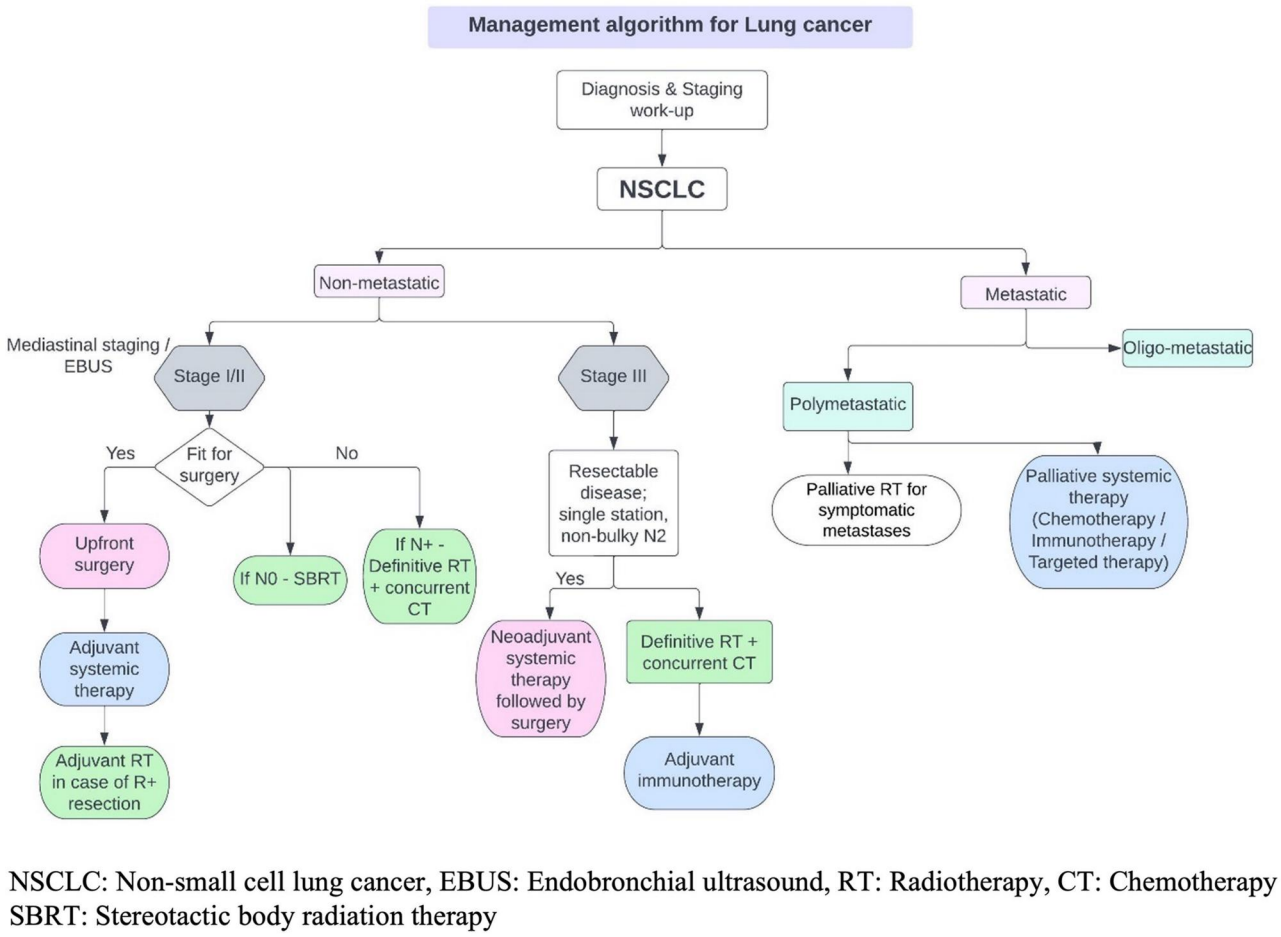
Management flowchart for non-small cell lung cancer (NSCLC).

**Figure 6.**
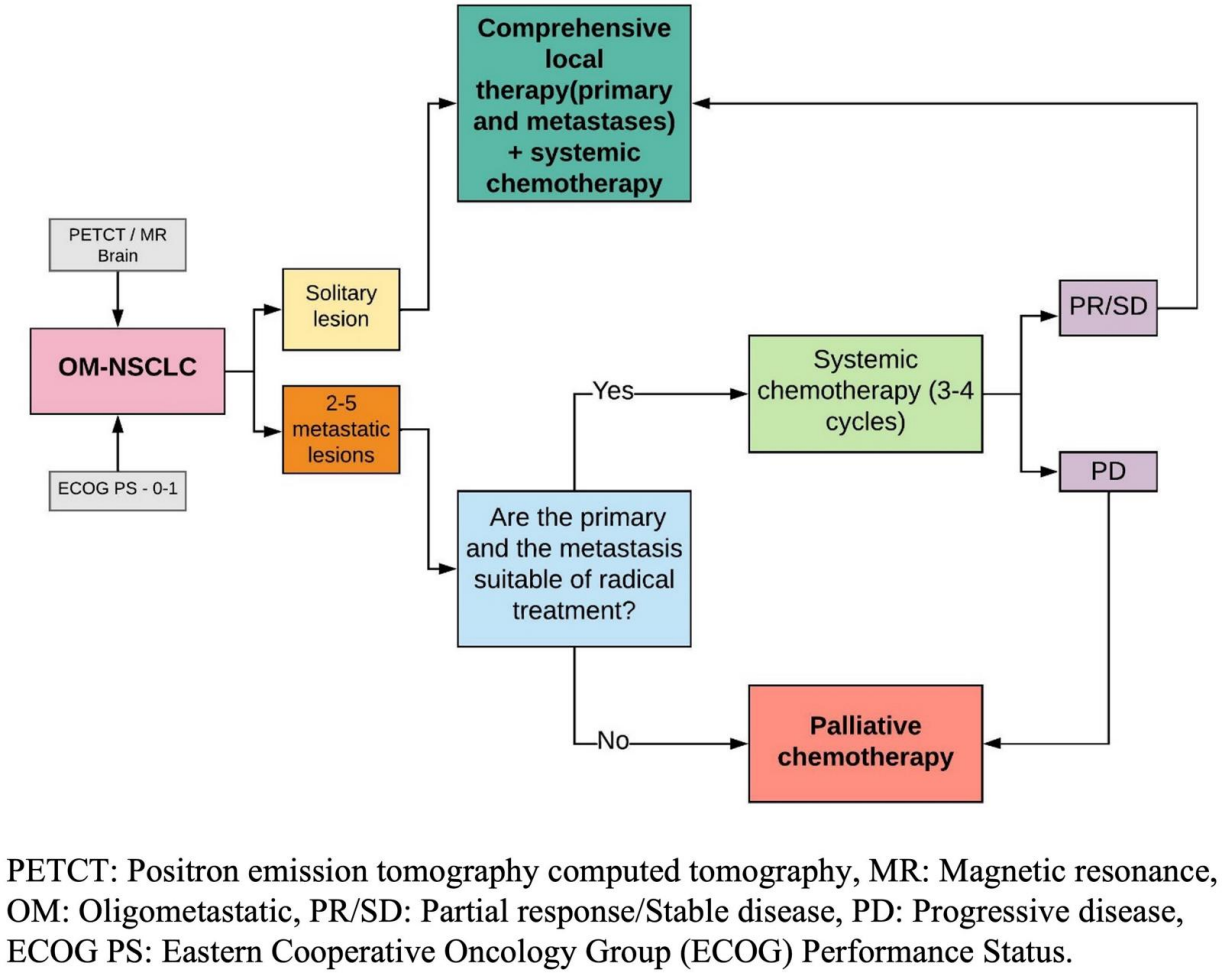
Management flowchart for oligometastatic non-small cell lung cancer (NSCLC).

Flowcharts for management of NSCLC are shown in Figures 5 and 6.

### SCLC

The contemporary management of limited stage SCLC (LS-SCLC) is definitive chemoradiotherapy using conventional or hyper-fractionated RT regimen with prophylactic cranial irradiation (PCI). Five-year survival in this setting is usually around 30% which is significantly lower than NSCLC.^81^ Extensive stage SCLC (ES-SCLC) is generally managed with palliative systematic chemotherapy with or without atezolizumab with a median survival of 10-12 months.^82^ The role of consolidative RT to primary disease with PCI has some survival benefit.^83^ The role of surgery in SCLC is limited to node negative, early stage (T1 or T2) tumours.^11^ Adjuvant therapy is a must after resection in SCLC.^84^

**Figure 7.**
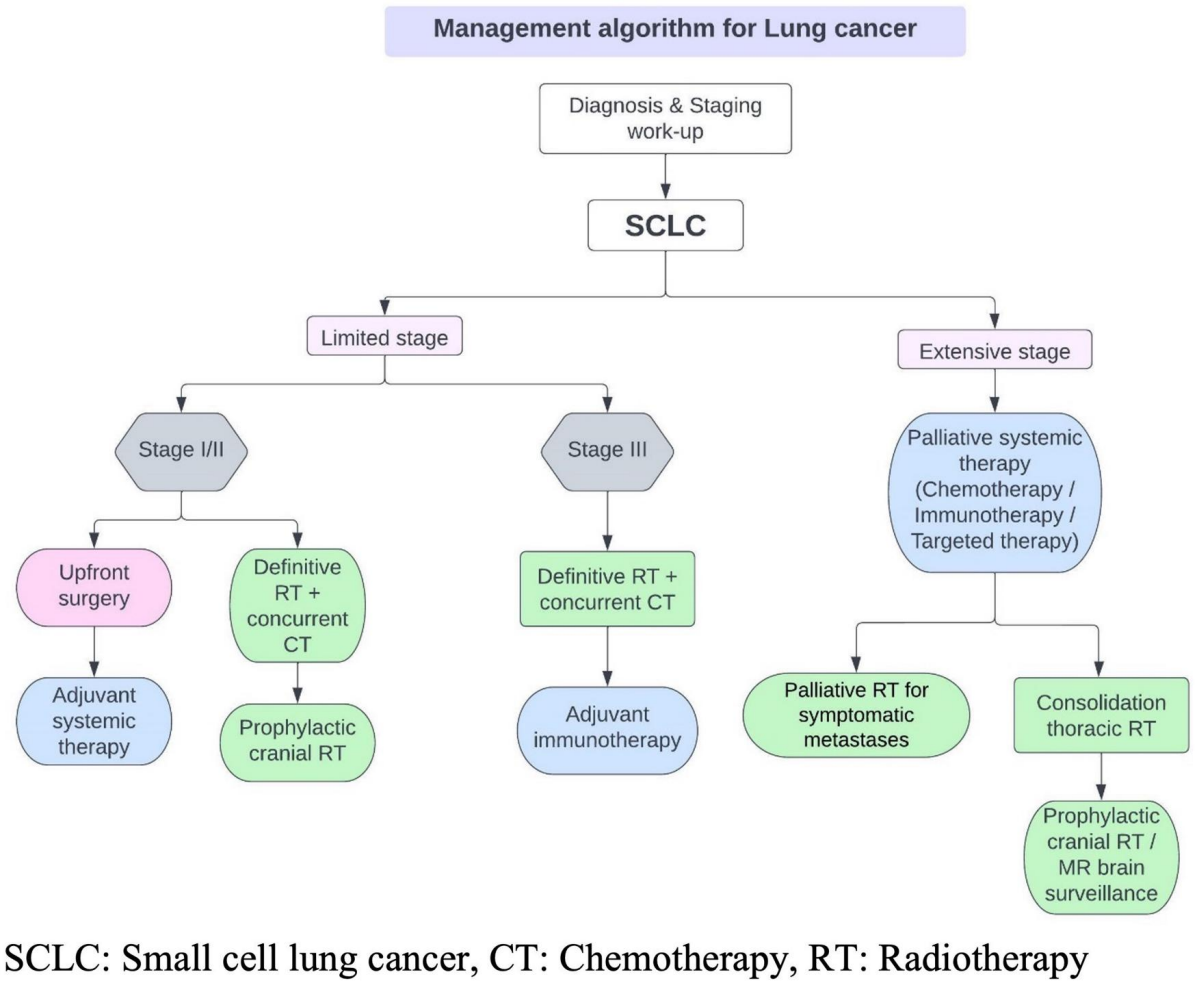
Management flowchart for small cell lung cancer (SCLC).

Flowchart for management of SCLC is shown in Figure 7.

### Pulmonary NET (other than SCLC)

For localized tumours, surgery is the treatment of choice for TC and AC. Adequate surgery yields 5-year survival of up to 90% in TC and 70% in AC. As most of these tumours are located in the central airways, surgery often involves a lobectomy with or without bronchoplastic techniques. Parenchymal preservation with oncologically complete resection is the goal for surgery in these tumours.^84^ In TC, initial endobronchial therapy in the form of laser or cryoablation or manual debulking may be used as a step towards parenchymal preservation.^85^ Occasionally, large central tumours may mandate a pneumonectomy for adequate clearance. Systematic hilar and mediastinal lymph nodal sampling or clearance is recommended in these tumours, since surgery is the mainstay of treatment.^84^

TC are purely surgical tumours and require no adjuvant therapy if completely resected. Node positive AC may benefit from adjuvant platinum, etoposide chemotherapy. Adjuvant therapy is a must after resection in LCNEC.^84^

For locally advanced TC and AC, surgery still remains the modality of choice. Unresectable TC are uncommon and every effort should be made to explore the possibility of surgery. If found impossible, they are best treated with Peptide Receptor Radionucleotide Therapy (PRRT); provided they have a good somatostatin uptake as seen on the DOTANOC PET scan.^84^ Locally advanced, unresectable AC and LCNEC are best treated with concurrent chemoradiation, as are unresectable TC with no DOTANOC uptake.^84^

The NCCN and European Neuroendocrine Tumour Society (ENETS) guidelines recommend the following treatment options in metastatic NETs:^86^^,^^87^

1. Watchful waiting in low grade, asymptomatic disease
2. Somatostatin analogues like octreotide and lanreotide in symptomatic (carcinoid syndrome) or those with octreotide positive scans
3. PRRT in tumours with DOTANOC uptake
4. Targeted therapeutic agents like Everolimus
5. Platinum and Etoposide based cytotoxic chemotherapy; or a combination of the above.

## Role of IR in lung tumour management (both primary and metastasis)

Various IR procedures for treatment of primary lung cancer and metastasis include CT/fluoroscopy guided radiofrequency ablation (RFA), microwave ablation and laser ablation, which are heat based procedures and cryoablation, which is a cold based ablative procedure. Thermal ablation is minimally invasive with high efficacy.^88^^,^^89^ Indications and contraindications of lung ablative procedures are enlisted in Table S2.^90^

Post procedure imaging should be done to evaluate adequacy of ablation or any post procedural complications, for example, check X-ray for pneumothorax and ultrasound screening for haemoperitoneum. Increase in size of the ablation scar, appearance of nodular, irregular, eccentric solid component in or at the margin of ablation zone or new contrast enhancement >15 Hounsfield unit (HU) on CT scan, and new FDG uptake in the scar >6 months after ablation are suggestive of local tumour progression as per the European Conference on Interventional Oncology (ECIO) and ESOI (European Society of Oncologic Imaging).^91^

## Follow-up imaging and role of IR in managing posttreatment complications

This section describes the imaging appearance of complications occurring after RT, chemotherapy, immunotherapy, and surgery. In addition, the role of IR in post-surgical complications have also been elucidated.

### Radiation induced lung injury (RILI)

This can be divided into radiation pneumonitis; the acute exudative phase within 6 months of RT completion, and the chronic fibrotic phase.^92^ Patterns of RILI and their appearance on CT include:

*Organizing pneumonia (OP) pattern*: occurs between 6 weeks to 10 months after RT completion and appears as patchy areas of GGO and consolidation with a waxing waning course often outside the RT field.^92^

*Modified conventional pattern:* most common pattern of RILI occurring after 6 months of RT presenting as focal area of consolidation, volume loss, and bronchiectasis within the RT field.^92^

*Mass-like pattern:* focal area of mass-like fibrosis without air-bronchogram and lacking straight margins, may be larger than the RT field, and is seen after 6 months of RT.^92^ A follow up CECT thorax can help differentiate post radiation injury from a recurrent disease.^93^

*Scar-like pattern:* seen as a thin linear opacity less than 1 cm in diameter with volume loss, occurring at the tumour site after 6 months of RT.^92^

### Post cytotoxic chemotherapy and targeted therapy complications

Drug-related pneumonitis is a known complication and most commonly occurs 5-10 days after 1-2 cycles of chemotherapy, early recognition/suspicion of which on a CECT thorax is essential for stopping the offending drug and confirming the resolution of lung changes on a follow up scan.^32^^,^^94^^,^^95^ Nonspecific interstitial pneumonia (NSIP), hypersensitivity pneumonitis (HP) and OP are the common CT patterns of post chemotherapy pneumonitis.^94^ Additional complication associated with cisplatin; a chemotherapeutic agent, could be arterial thromboembolism.^96^ EGFR and ALK inhibitors can show a variety of lung injury patterns on CT such as, OP, NSIP, HP, diffuse alveolar damage (DAD), and simple pulmonary eosinophilia.^97^ CT appearance of these findings are as follows:

*OP:* peripheral, subpleural or peribronchial consolidation with/without nodules are usually seen. Central GGO surrounded by peripheral consolidation known as “Atoll’s sign”, can be seen.^93^^,^^98^

*NSIP:* patchy GGO in early stages with basal fibrosis and traction bronchiectasis in the later stages.^98^

*HP:* the acute stage of the disease is marked by bilateral symmetrical GGO, mainly affecting the middle and lower lobes, with or without peri-bronchovascular involvement. In the subacute stage, there is a combination of GGO and air-space nodules. In the chronic stages, fibrosis occurs, with a tendency to affect the upper lobes of the lungs.^98^

*DAD:* bilateral GGO, which may appear either scattered or diffuse across the lungs.^98^

*Pulmonary eosinophilia:* peripheral areas of consolidation, creating an appearance known as “reverse pulmonary edema.”^98^

### Post immunotherapy complications

Various complications after immunotherapy and their imaging features are as follows:

*Pneumonitis*: Commonest patterns are OP and GGO; however, (NSIP), HP, acute interstitial pneumonia (AIP)-acute respiratory distress syndrome (ARDS), and simple pulmonary eosinophilia patterns have also been described.^99^

*Sarcoid-like reactions:* Typically present with mediastinal and bilateral hilar lymphadenopathy along with bilateral upper lobe nodules in the peribronchovascular or perilymphatic distribution.^99^

*Cardiovascular-related complications:* Myocarditis; subendocardial-transmural, subepicardial, mid-myocardial, and diffuse patterns of late gadolinium enhancement on MRI, pericarditis, pericardial effusion, tamponade, and vasculitis (including temporal arteritis) are the various cardiovascular-related complications.^99^

*Colitis:* Diffuse (pancolitis) or segmental colonic mural thickening (segmental colitis) may be seen on CECT, associated with diverticulosis. Imaging is required to rule out complications such as bowel perforation, obstruction, and toxic megacolon.^99^

*Hepatitis:* Mild hepatomegaly, periportal oedema, and periportal lymphadenopathy may be seen on CECT in severe cases, whereas, imaging may normal in mild cases.^99^

*Pancreatitis:* CECT findings include enlarged-oedematous pancreas with peripancreatic fat stranding and reduced enhancement.^99^

*Cholangitis:* CT shows gall bladderwall thickening and periportal oedema.^99^

*Adrenalitis:* Enlarged adrenal glands with FDG avidity can be seen on PET-CECT.^99^

*Thyroiditis:* US shows diffusely hypoechoic and heterogeneous thyroid.^99^

*Hypophysitis:* MRI findings of enlarged pituitary gland can precede new onset hypopituitarism and clinical features.^99^

*Neurological complications:* Encephalitis shows bilateral or unilateral hyperintensity in the mesial temporal lobes/lobe on T2WI and fluid-attenuated inversion-recovery (FLAIR) MRI images. In meningitis, MRI may reveal leptomeningeal and/or pachymeningeal enhancement, increased T2 signal intensity in the meninges, or nerve root enhancement. T2 hyperintense lesions with focal cord enhancement may be seen in the spinal cord in myelitis. Demyelinating diseases often present with acute features such as contrast enhancement or T2 hyperintensity in either spinal cord or optic nerve, or two or more brain regions (eg, periventricular, cortical, juxtacortical, infratentorial), with symptoms corresponding to the neuroanatomic location.^99^

### Radiation recall pneumonitis

Phenomenon in which new onset sharply demarcated GGO is seen on CT thorax in a prior radiation field, manifesting years after RT when traditional cytotoxic drugs, tyrosine kinase inhibitor or immune checkpoint inhibitors are administered.^93^^,^^99^

### Post-surgical complications

There are different types of post-surgical complications -collapse, mediastinal hematoma, haemothorax, pneumothorax, pulmonary oedema, chylothorax, acute respiratory distress syndrome (ARDS), broncho-pleural fistula, empyema, and pericardial effusion.^100^ IR has a major role in post-surgical management by placing a catheter or draining the collection percutaneously under image guidance (ultrasound/CT), thus averting a major surgery. Choice of imaging modality depends upon the location of collection and its content, for example, air containing collection needs CT guidance, haemothorax, pneumothorax, or empyema can be managed by ultrasound guided drainage. Chylothorax can be diagnosed with lymphangiography and managed with embolization of lymphatic leak.

**Figure 8.**
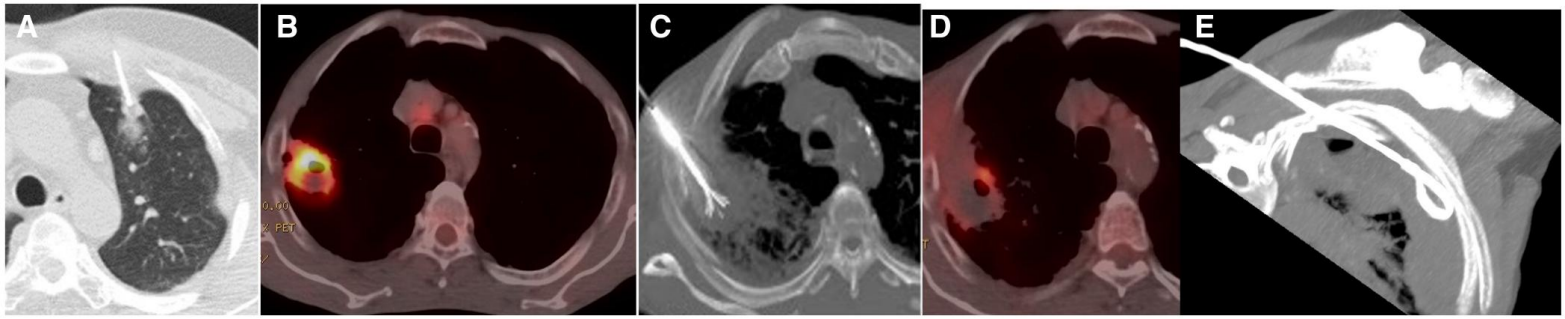
Role of interventional radiology in lung cancer management. (A) CT guided biopsy from left upper lobe lung adenocarcinoma done with oblique approach lateral to the left internal mammary artery with biopsy needle tip within the lesion. (B) Pre radiofrequency ablation (RFA) FDG PET CT shows metabolic activity within the lung cancer. (C) Position of RFA electrode within the lung lesion shown in B. (D) Post RFA FDG PET CT showing residual activity only at the periphery of the lesion in B. (E) Right pleural effusion after surgery for lung cancer with CT guided pigtail in situ.

Role of IR in lung cancer management is shown in Figure 8.

## Management of recurrent disease

Management of any recurrences either locoregional or distant requires rigorous multidisciplinary coordination. Usually post-operative, radiation naive locoregional recurrences are considered to be patho-physiologically same as originally nonresectable stage IIIA and IIIB diseases which can be managed with definitive RT with or without chemotherapy with good clinical control.^101^ Salvage surgery is a good option in fit patients with locoregional progression of an unresectable Stage III tumours initially managed with definitive CTRT with a median OS ranging from 13 to 76 months. Pneumonectomy is usually the most common surgical approach in salvage setting.^102^ Distant recurrences are either oligo-metastatic (1-5 metastasis) or poly-metastatic (>5 mets). Poly-metastatic recurrences are usually managed with palliative systemic chemotherapy or immunotherapies and targeted therapies as appropriate. Whereas in oligo-recurrence cases with fewer metastasis, metastatectomy or ablative radiotherapy along with systemic therapy has become the standard of care.^103^ Oligo-progression in patients with stage IV NSCLC developed while receiving systemic therapy is usually challenged with alternate systemic therapies. Targeting oligo-progressive metastasis with ablative radiotherapy may have some benefit in progression free survival.^104^^,^^105^

## Role of artificial intelligence (AI)

There has been an upsurge in radiomics and deep learning related research in cancer care ranging from risk stratification, screening and diagnosis, to prediction of molecular mutations, treatment response, and survival outcomes, though deployable model is still not available.^106^ Developed AI application should not only be validated and tested, but should also follow certain consensus guidelines laid down by the international bodies, for example,

Standards for Reporting Diagnostic Accuracy (STARD) statement, Transparent Reporting of a multivariable prediction model for Individual Prognosis Or Diagnosis (TRIPOD) statement, and radiomics quality score (RQS).^107–110^

Few of the many AI based studies pertaining to lung cancer include: (1) Screening recommendation based on risk stratification.^111^^,^^112^ (2) Prediction of EGFR and anaplastic ALK mutations, and PD-L1 expression.^113–117^ (3) Prediction of response to immunotherapy and differentiation of true progression from pseudoprogression after immunotherapy using delta radiomics.^118^^,^^119^ (4) Prediction of survival outcomes in NSCLC and SCLC using baseline CECT.^120^^,^^121^

Synoptic reporting templates 1 and 2 on “pre-treatment Lung Cancer Imaging—Reporting and Data System (LCI-RADS)” and “post-therapy Lung Cancer Imaging—Reporting and Data System (pLCI-RADS)” are attached as supplementary materials S3 and S4, respectively.^32^

## Conclusion

Understanding imaging patterns, selecting the right imaging modalities, and conducting a thorough work-up are essential for optimal lung cancer management. This article offers flowcharts based on current guidelines and recommendations, providing valuable tools for effective lung cancer care. Additionally, it includes discussions on the role of interventional radiology and strategies for managing recurrent lung cancer. These insights can assist clinicians in making informed decisions, ensuring comprehensive and effective patient care. The combination of these elements supports clinicians in navigating complex cases and optimizing treatment outcomes for lung cancer patients.

## Supplementary Material

tzaf013_Supplementary_Data

## References

[1] Chakrabarty N , Mahajan A, Patil V, Noronha V, Prabhash K. Imaging of brain metastasis in non-small-cell lung cancer: indications, protocols, diagnosis, post-therapy imaging, and implications regarding management. Clin Radiol. 2023;78:175-186. 36503631 10.1016/j.crad.2022.09.134

[2] Sung H , Ferlay J, Siegel RL, et al Global cancer statistics 2020: GLOBOCAN estimates of incidence and mortality worldwide for 36 cancers in 185 countries. CA Cancer J Clin. 2021;71:209-e249. 33538338 10.3322/caac.21660

[3] Ganti AK , Klein AB, Cotarla I, Seal B, Chou E. Update of Incidence, Prevalence, Survival, and Initial Treatment in Patients With Non–Small Cell Lung Cancer in the US. JAMA Oncol. 2021;7:1824-1832. 34673888 10.1001/jamaoncol.2021.4932 PMC8532041

[4] Lung neuroendocrine (carcinoid tumors): Epidemiology, risk factors, classification, histology, diagnosis, and staging. Accessed December 10, 2023. https://www.uptodate.com/contents/lung-neuroendocrine-carcinoid-tumors-epidemiology-risk-factors-classification-histology-diagnosis-and-staging#:∼:text=Lung%20neuroendocrine%20tumors%20(NETs)%20account,NETs%20%5B1%2D4%5D

[5] Pelosi G , Sonzogni A, Harari S, et al Classification of pulmonary neuroendocrine tumors: new insights. Transl Lung Cancer Res. 2017;6:513-529. 29114468 10.21037/tlcr.2017.09.04 PMC5653522

[6] IARC Working Group on the Evaluation of Carcinogenic Risks to Humans, World Health Organization, International Agency for Research on Cancer. *Tobacco Smoke and Involuntary Smoking*. IARC; 2004. PMC4781536 15285078

[7] Zou K , Sun P, Huang H, et al Etiology of lung cancer: evidence from epidemiologic studies. J Natl Cancer Center. 2022;2:216-225. 39036545 10.1016/j.jncc.2022.09.004 PMC11256564

[8] Khuder SA. Effect of cigarette smoking on major histological types of lung cancer: a meta-analysis. Lung Cancer. 2001;31:139-148. 11165392 10.1016/s0169-5002(00)00181-1

[9] Veglia F , Vineis P, Overvad K, et al Occupational exposures, environmental tobacco smoke, and lung cancer. Epidemiology. 2007;18:769-775. 18062064 10.1097/ede.0b013e318142c8a1

[10] Planchard D , Loriot Y, Goubar A, et al Differential expression of biomarkers in men and women. Semin Oncol. 2009;36:553-565. 10.1053/j.seminoncol.2009.09.004 19995647

[11] NCCN Clinical Practice Guidelines in Oncology (NCCN Guidelines),version 3. 2024. Accessed June 28, 2024. www.nccn.org/professionals/physician_gls/pdf/sclc.pdf

[12] Kanaji N , Watanabe N, Kita N, et al Paraneoplastic syndromes associated with lung cancer. World J Clin Oncol. 2014;5:197-223. 25114839 10.5306/wjco.v5.i3.197 PMC4127595

[13] Gregg JP , Li T, Yoneda KY. Molecular testing strategies in non-small cell lung cancer: optimizing the diagnostic journey. Transl Lung Cancer Res. 2019;8:286-301. 31367542 10.21037/tlcr.2019.04.14 PMC6626860

[14] NCCN Clinical Practice Guidelines in Oncology (NCCN Guidelines), version 7. 2024. Accessed June 28, 2024. https://www.nccn.org/professionals/physician_gls/pdf/nscl.pdf

[15] Raman R , Ramamohan V, Rathore A, Jain D, Mohan A, Vashistha V. Prevalence of highly actionable mutations among Indian patients with advanced non-small cell lung cancer: A systematic review and meta-analysis. Asia Pac J Clin Oncol. 2023;19:158-171. 35634796 10.1111/ajco.13802

[16] Dingemans AC , Früh M, Ardizzoni A, et al Small-cell lung cancer: ESMO Clinical Practice Guidelines for diagnosis, treatment and follow-up. Ann Oncol. 2021;32:839-853. 33864941 10.1016/j.annonc.2021.03.207 PMC9464246

[17] Remon J , Soria J-C, Peters S. Early and locally advanced non-small-cell lung cancer: an update of the ESMO Clinical Practice Guidelines focusing on diagnosis, staging, systemic and local therapy. Ann Oncol. 2021;32:1637-1642. 34481037 10.1016/j.annonc.2021.08.1994

[18] Schneider BJ , Ismaila N, Aerts J, et al Lung cancer surveillance after definitive curative-intent therapy: ASCO Guideline. J Clin Oncol. 2020;38:753-766. 31829901 10.1200/JCO.19.02748

[19] Aberle DR , Adams AM, Berg CD, et al Reduced lung-cancer mortality with low-dose computed tomographic screening. N Engl J Med. 2011;365:395-409. 21714641 10.1056/NEJMoa1102873 PMC4356534

[20] Wang C , Shao J, Song L, et al Persistent increase and improved survival of stage I lung cancer based on a large-scale real-world sample of 26,226 cases. Chin Med J (Engl). 2023;136:1937-1948. 37394562 10.1097/CM9.0000000000002729 PMC10431578

[21] Jonas DE , Reuland DS, Reddy SM, et al Screening for lung cancer with low-dose computed tomography: updated evidence report and systematic review for the US Preventive Services Task Force. JAMA. 2021;325:971-987. 33687468 10.1001/jama.2021.0377

[22] Guidelines for lung cancer screening. Accessed August 11, 2024. https://www.uptodate.com/contents/image?imageKey=PULM%2F64078

[23] Lung-RADS. Accessed August 11, 2024. https://www.acr.org/-/media/ACR/Files/RADS/Lung-RADS/Lung-RADS-2022.pdf

[24] Argentieri G , Valsecchi C, Petrella F, et al Implementation of the 9th TNM for lung cancer: practical insights for radiologists. Eur Radiol. 2025; 10.1007/s00330-024-11345-8 PMC12165876 39825171

[25] Erasmus LT , Strange CD, Ahuja J, et al Imaging of Lung Cancer Staging: TNM 9 Updates. Semin Ultrasound CT MR S0887-2171:00045–3, 2024. 10.1053/j.sult.2024.07.005 39069273

[26] Asamura H , Nishimura KK, Giroux DJ, et al IASLC Lung Cancer Staging Project: The new database to inform revisions in the ninth edition of the TNM classification of lung cancer. J Thorac Oncol. 2023;18:564-575. 36773775 10.1016/j.jtho.2023.01.088

[27] Detterbeck FC , Woodard GA, Bader AS, et al The proposed ninth edition TNM classification of lung cancer. Chest. 2024;166:882-895. 10.1016/j.chest.2024.05.026 Epub 2024 Jun 15. 38885896

[28] Rami-Porta R , Nishimura KK, Giroux DJ, et al The International Association for the Study of Lung Cancer Lung Cancer Staging Project: proposals for revision of the TNM Stage Groups in the Forthcoming (Ninth) Edition of the TNM Classification for Lung Cancer. J Thorac Oncol. 2024;19:1007-1027. 38447919 10.1016/j.jtho.2024.02.011

[29] Huang J , Osarogiagbon RU, Giroux DJ, et al The International Association for the Study of Lung Cancer Staging Project for Lung Cancer: Proposals for the Revision of the N Descriptors in the Forthcoming Ninth Edition of the TNM Classification for Lung Cancer. J Thorac Oncol. 2024;19:766-785. 37866624 10.1016/j.jtho.2023.10.012 PMC12323887

[30] El-Sherief AH , Lau CT, Wu CC, Drake RL, Abbott GF, Rice TW. International association for the study of lung cancer (IASLC) lymph node map: radiologic review with CT illustration. Radiographics. 2014;34:1680-1691. 25310423 10.1148/rg.346130097

[31] Li HY , Wang YY, Liu H, et al [The ninth edition of TNM staging for lung cancer: precise staging for precise diagnosis and treatment]. Zhonghua Wai Ke Za Zhi. 2024;62:537-542. Chinese. 10.3760/cma.j.cn112139-20231210-00262. 38682624

[32] Chakrabarty N , Mahajan A. Pre-treatment “Lung Cancer Imaging—Reporting and Data System” (LCI-RADS) and “Post-therapy Lung Cancer Imaging—Reporting and Data System” (pLCI-RADS): a narrative review of comprehensive synoptic reporting formats for lung cancer imaging. Cancer Res Stat Treat. 2022;5:734-742.

[33] Bhalla AS , Das A, Naranje P, Irodi A, Raj V, Goyal A. Imaging protocols for CT chest: a recommendation. Indian J Radiol Imaging. 2019;29:236-246. 10.4103/ijri.IJRI_34_19. 31741590 PMC6857267

[34] Lambe G , Durand M, Buckley A, Nicholson S, McDermott R. Adenocarcinoma of the lung: from BAC to the future. Insights Imaging. 2020;11:69. 32430670 10.1186/s13244-020-00875-6 PMC7237554

[35] Han X , Fan J, Gu J, et al CT features associated with EGFR mutations and ALK positivity in patients with multiple primary lung adenocarcinomas. Cancer Imaging. 2020;20:51. 32690092 10.1186/s40644-020-00330-1 PMC7372851

[36] Han X , Fan J, Li Y, et al Value of CT features for predicting EGFR mutations and ALK positivity in patients with lung adenocarcinoma. Sci Rep. 2021;11:5679. 10.1038/s41598-021-83646-7 33707479 PMC7952563

[37] Hollings N , Shaw P. Diagnostic imaging of lung cancer. Eur. Respir. J. 2002;19:722-742. 11999004 10.1183/09031936.02.00280002

[38] Chong S , Lee KS, Chung MJ, Han J, Kwon OJ, Kim TS. Neuroendocrine tumors of the lung: clinical, pathologic, and imaging findings. Radiographics. 2006;26:41-57. 16418242 10.1148/rg.261055057

[39] Sun X , Zeng B, Tan X, Chen Z, Pan X, Jiang L. Invasive mucinous adenocarcinoma of the lung: clinicopathological features, ^18^F-FDG PET/CT findings, and survival outcomes. Ann Nucl Med. 2023;37:198-207. 36538165 10.1007/s12149-022-01816-7

[40] Lamarca A , Pritchard DM, Westwood T, et al 68Gallium DOTANOC-PET imaging in lung carcinoids: impact on patients’ management. Neuroendocrinology. 2018;106:128-138. 28399530 10.1159/000472717

[41] Le Rhun E , Weller M, Brandsma D, et al EANOeESMO Clinical Practice Guidelines for diagnosis, treatment and follow-up of patients with leptomeningeal metastasis from solid tumours. Ann Oncol. 2017;28:iv84-iv99. 28881917 10.1093/annonc/mdx221

[42] Singh SK , Leeds NE, Ginsberg LE. MR imaging of leptomeningeal metastases: comparison of three sequences. AJNR Am J Neuroradiol. 2002;23:817-821. 12006284 PMC7974747

[43] Wever W , Ceyssens S, Mortelmans L, et al Additional value of PET-CT in the staging of lung cancer: comparison with CT alone, PET alone and visual correlation of PET and CT. Eur Radiol. 2007;17:23-32. 16683115 10.1007/s00330-006-0284-4

[44] Suh YJ , Park CM, Han K, et al Utility of FDG PET/CT for preoperative staging of non–small cell lung cancers manifesting as subsolid nodules with a solid portion of 3 cm or smaller. AJR Am J Roentgenol. 2020;214:514-523. 31846374 10.2214/AJR.19.21811

[45] Sung YM , Lee KS, Kim BT, et al 18F-FDG PET versus 18F-FDG PET/CT for adrenal gland lesion characterization: a comparison of diagnostic efficacy in lung cancer patients. Korean J Radiol. 2008;9:19-28. 18253072 10.3348/kjr.2008.9.1.19 PMC2627169

[46] Park SY , Park BK, Kim CK. The value of adding 18F-FDG PET/CT to adrenal protocol CT for characterizing adrenal metastasis (≥10 mm) in oncologic patients. AJR Am J Roentgenol. 2014;202:W153-W160. 24450697 10.2214/AJR.13.10873

[47] Krüger S , Mottaghy MF, Buck KA, et al Brain metastasis in lung cancer. Nuklearmedizin. 2011;50:101-106. 21165538 10.3413/Nukmed-0338-10-07

[48] Ferrigno D , Buccheri G. Cranial computed tomography as a part of the initial staging procedures for patients with non-small-cell lung cancer. Chest. 1994;106:1025-1029. 7924469 10.1378/chest.106.4.1025

[49] Rodrigues M , Stark H, Rendl G, et al Diagnostic performance of [18F] FDG PET-CT compared to bone scintigraphy for the detection of bone metastases in lung cancer patients. Q J Nucl Med Mol Imaging. 2016;60:62-68. 26844431

[50] Zidan MA , Hassan RS, El Noueam KI, et al Brain metastases assessment by FDG-PET/CT: can it eliminate the necessity for dedicated brain imaging? Egypt J Radiol Nucl Med. 2020;51:223.

[51] Kitajima K , Nakamoto Y, Okizuka H, et al Accuracy of whole-body FDGPET/CT for detecting brain metastases from non-central nervous system tumors. Ann Nucl Med. 2008;22:595-e602. 18756362 10.1007/s12149-008-0145-0

[52] Li Y , Jin G, Su D. Comparison of gadolinium-enhanced MRI and 18FDG PET/PET-CT for the diagnosis of brain metastases in lung cancer patients: a meta-analysis of 5 prospective studies. Oncotarget. 2017;8:35743-35749. 28415747 10.18632/oncotarget.16182 PMC5482613

[53] Schoenmaekers J , Hofman P, Bootsma G, et al Screening for brain metastases in patients with stage III non-small-cell lung cancer, magnetic resonance imaging or computed tomography? A prospective study. Eur J Cancer. 2019;115:88-96. 31129385 10.1016/j.ejca.2019.04.017

[54] Khosla A. Brain Metastasis Imaging. Medscape. Accessed October 1, 2024 at https://emedicine.medscape.com/article/338239-overview#a3.

[55] DiBardino DM , Yarmus LB, Semaan RW. Transthoracic needle biopsy of the lung. J Thorac Dis. 2015;7:S304-S316. 26807279 10.3978/j.issn.2072-1439.2015.12.16 PMC4700361

[56] Lentz RJ , Frederick-Dyer K, Planz VB, et al Navigational bronchoscopy vs CT scan-guided transthoracic needle biopsy for the diagnosis of indeterminate lung nodules: protocol and rationale for the navigation endoscopy to reach indeterminate lung nodules vs transthoracic needle aspiration, a randomized controlled study multicenter randomized trial. Chest Pulmon. 2024;2:100050. 10.1016/j.chpulm.2024.100050 PMC13418960 42548449

[57] Kothary N , Lock L, Sze DY, et al Computed tomography–guided percutaneous needle biopsy of pulmonary nodules: impact of nodule size on diagnostic accuracy. Clin Lung Cancer. 2009;10:360-363. 19808195 10.3816/CLC.2009.n.049

[58] Ng YL , Patsios D, Roberts H, et al CT-guided percutaneous fine-needle aspiration biopsy of pulmonary nodules measuring 10 mm or less. Clin Radiol. 2008;63:272-277. 18275867 10.1016/j.crad.2007.09.003

[59] Vachani A , Zhou M, Ghosh S, et al Complications after transthoracic needle biopsy of pulmonary nodules: a population-level retrospective cohort analysis. J Am Coll Radiol. 2022;19:1121-1129. 35738412 10.1016/j.jacr.2022.04.010

[60] Lin NU , Lee EQ, Aoyama H, et al Response assessment criteria for brain metastases: proposal from the RANO group. Lancet Oncol. 2015;16:e270-e278. 26065612 10.1016/S1470-2045(15)70057-4

[61] Veltri A , Bargellini I, Giorgi L, Almeida PAMS, Akhan O. CIRSE guidelines on percutaneous needle biopsy (PNB). Cardiovasc Intervent Radiol. 2017;40:1501-1513. 28523447 10.1007/s00270-017-1658-5

[62] Gupta S , Krishnamurthy S, Broemeling LD, et al Small (B 2-cm) subpleural pulmonary lesions: short- versus long-needle-path CT-guided biopsy—comparison of diagnostic yields and complications. Radiology. 2005;234:631-637. 15673500 10.1148/radiol.2342031423

[63] Folch EE , Pritchett MA, Nead MA, et al Electromagnetic navigation bronchoscopy for peripheral pulmonary lesions: one-year results of the prospective, multicenter NAVIGATE study. J Thorac Oncol. 2019;14:445-458. 30476574 10.1016/j.jtho.2018.11.013

[64] Aboudara M , Roller L, Rickman O, et al Improved diagnostic yield for lung nodules with digital tomosynthesis‐corrected navigational bronchoscopy: initial experience with a novel adjunct. Respirology. 2020;25:206-213. 31265204 10.1111/resp.13609

[65] Low SW , Lentz RJ, Chen H, et al Shape-sensing robotic-assisted bronchoscopy vs digital tomosynthesis-corrected electromagnetic navigation bronchoscopy: a comparative cohort study of diagnostic performance. Chest. 2023;163:977-984. 36441041 10.1016/j.chest.2022.10.019

[66] Chalian H , Töre HG, Horowitz JM, Salem R, Miller FH, Yaghmai V. Radiologic assessment of response to therapy: comparison of RECIST Versions 1.1 and 1.0. Radiographics. 2011;31:2093-2105. 22084190 10.1148/rg.317115050

[67] Persigehl T , Lennartz S, Schwartz LH. iRECIST: how to do it. Cancer Imaging. 2020;20:2. 31900236 10.1186/s40644-019-0281-x PMC6942293

[68] Aide N , Hicks RJ, Le Tourneau C, Lheureux S, Fanti S, Lopci E. FDG PET/CT for assessing tumour response to immunotherapy: report on the EANM symposium on immune modulation and recent review of the literature. Eur J NuclMed Mol Imaging. 2019;46:238-250. 10.1007/s00259-018-4171-4 PMC6267687 30291373

[69] Altorki N , Wang X, Kozono D, et al Lobar or sublobar resection for peripheral stage IA non-small-cell lung cancer. N Engl J Med. 2023;388:489-498. 10.1056/NEJMoa2212083 36780674 PMC10036605

[70] Chang JY , Mehran RJ, Feng L, et al Stereotactic ablative radiotherapy for operable stage I non-small-cell lung cancer (revised STARS): long-term results of a single-arm, prospective trial with prespecified comparison to surgery. Lancet Oncol. 2021;22:1448-1457. 10.1016/S1470-2045(21)00401-0 34529930 PMC8521627

[71] Douillard JY , Rosell R, De Lena M, et al Adjuvant vinorelbine plus cisplatin versus observation in patients with completely resected stage IB-IIIA non-small-cell lung cancer (Adjuvant Navelbine International Trialist Association [ANITA]): a randomised controlled trial. Lancet Oncol. 2006;7:719-727. 10.1016/S1470-2045(06)70804-X 16945766

[72] Butts CA , Ding K, Seymour L, et al Randomized phase III trial of vinorelbine plus cisplatin compared with observation in completely resected stage IB and II non-small-cell lung cancer: updated survival analysis of JBR-10. J Clin Oncol. 2010;28:29-34. 10.1200/JCO.2009.24.0333 19933915 PMC2799232

[73] Pignon JP , Tribodet H, Scagliotti GV, et al Lung adjuvant cisplatin evaluation: a pooled analysis by the LACE Collaborative Group. J Clin Oncol. 2008;26:3552-3559. 10.1200/JCO.2007.13.9030 18506026

[74] Pisters K , Kris MG, Gaspar LE, et al Adjuvant systemic therapy and adjuvant radiation therapy for stage I-IIIA completely resected non-small-cell lung cancer: ASCO Guideline Rapid Recommendation Update. J Clin Oncol. 2022;40:1127-1129. 10.1200/JCO.22.00051 35167335

[75] Wu YL , Tsuboi M, He J, et al Osimertinib in resected *EGFR*-mutated non-small-cell lung cancer. N Engl J Med. 2020;383:1711-1723. 10.1056/NEJMoa2027071 32955177

[76] Felip E , Altorki N, Zhou C, et al Adjuvant atezolizumab after adjuvant chemotherapy in resected stage IB-IIIA non-small-cell lung cancer (IMpower010): a randomised, multicentre, open-label, phase 3 trial. Lancet. 2021;398:1344-1357. 10.1016/S0140-6736(21)02098-5 34555333

[77] Wang EH , Corso CD, Rutter CE, et al Postoperative radiation therapy is associated with improved overall survival in incompletely resected stage II and III non-small-cell lung cancer. J Clin Oncol. 2015;33:2727-2734. 10.1200/JCO.2015.61.1517 26101240

[78] Urvay SE , Yucel B, Erdis E, et al Prognostic factors in stage III non-small-cell lung cancer patients. Asian Pac J Cancer Prev. 2016;17:4693-4697. 10.22034/apjcp.2016.17.10.4693 27893199 PMC5454619

[79] Antonia SJ , Villegas A, Daniel D, et al Durvalumab after chemoradiotherapy in stage III non-small-cell lung cancer. N Engl J Med. 2017;377:1919-1929. 10.1056/NEJMoa1709937 28885881

[80] Chen R , Manochakian R, James L, et al Emerging therapeutic agents for advanced non-small cell lung cancer. J Hematol Oncol. 2020;13:58. 10.1186/s13045-020-00881-7 32448366 PMC7245927

[81] Petrelli F , Ghidini A, Cabiddu M, et al Addition of radiotherapy to the primary tumour in oligometastatic NSCLC: a systematic review and meta-analysis. Lung Cancer. 2018;126:194-200. 10.1016/j.lungcan.2018.11.017 30527187

[82] Faivre-Finn C , Snee M, Ashcroft L, et al Concurrent once-daily versus twice-daily chemoradiotherapy in patients with limited-stage small-cell lung cancer (CONVERT): an open-label, phase 3, randomised, superiority trial. Lancet Oncol. 2017;18:1116-1125. 28642008 10.1016/S1470-2045(17)30318-2 PMC5555437

[83] Horn L , Mansfield AS, Szczęsna A, et al First-line atezolizumab plus chemotherapy in extensive-stage small-cell lung cancer. N Engl J Med. 2018;379:2220-2229. 30280641 10.1056/NEJMoa1809064

[84] Slotman BJ , van Tinteren H, Praag JO, et al Use of thoracic radiotherapy for extensive stage small-cell lung cancer: a phase 3 randomised controlled trial. Lancet. 2015;385:36-42. 25230595 10.1016/S0140-6736(14)61085-0

[85] Gosain R , Mukherjee S, Yendamuri S, et al Management of typical and atypical pulmonary carcinoids based on different established guidelines. Cancers (Basel). 2018;10:510. 30545054 10.3390/cancers10120510 PMC6315766

[86] Reuling EMBP , Dickhoff C, Plaisier PW, et al Endobronchial treatment for bronchial carcinoid: patient selection and predictors of outcome. Respiration. 2018;95:220-227. 29433123 10.1159/000484984 PMC6067647

[87] NCCN Practice Guidelines in Oncology. Neuroendocrine and Adrenal Tumors. Version: 1. 2024. Accessed June 28, 2024.

[88] Caplin ME , Baudin E, Ferolla P, et al Pulmonary neuroendocrine (carcinoid) tumors: European Neuroendocrine Tumor Society expert consensus and recommendations for best practice for typical and atypical pulmonary carcinoids. Ann Oncol. 2015;26:1604-1620. 25646366 10.1093/annonc/mdv041

[89] Smith SL , Jennings PE. Lung radiofrequency and microwave ablation: A review of indications, techniques and postprocedural imaging appearances. Br J Radiol. 2015;88:20140598. 25465192 10.1259/bjr.20140598 PMC4614249

[90] Matsui Y , Hiraki T, Gobara H, et al Long-term survival following percutaneous radiofrequency ablation of colorectal lung metastases. J Vasc Interv Radiol. 2015;26:303-310; quiz 311. 25612808 10.1016/j.jvir.2014.11.013

[91] Venturini M , Cariati M, Marra P, Masala S, Pereira PL, Carrafiello G. CIRSE standards of practice on thermal ablation of primary and secondary lung tumours. Cardiovasc Intervent Radiol. 2020;43:667-683. 32095842 10.1007/s00270-020-02432-6

[92] Strange TA , Erasmus LT, Ahuja J, et al Spectrum of imaging patterns of lung cancer following radiation therapy. Diagnostics (Basel). 2023;13:3283. 37892105 10.3390/diagnostics13203283 PMC10606648

[93] Sridhar S , Kanne JP, Henry TS, Revels JW, Gotway MB, Ketai LH. Medication-induced pulmonary injury: a scenario- and pattern-based approach to a perplexing problem. Radiographics. 2022;42:38-55. 34826256 10.1148/rg.210146

[94] Murphy DJ , Mayoral M, Larici AR, et al Imaging follow-up of nonsurgical therapies for lung cancer: AJR expert panel narrative review. Am J Roentgenol. 2023;221:409-424. 10.2214/AJR.23.29104 37095669 PMC11037936

[95] Maas M , Beets-Tan R, Gaubert J-Y, et al Follow-up after radiological intervention in oncology: ECIO-ESOI evidence and consensus-based recommendations for clinical practice. Insights Imaging. 2020;11:83. 32676924 10.1186/s13244-020-00884-5 PMC7366866

[96] Torrisi JM , Schwartz LH, Gollub MJ, Ginsberg MS, Bosl GJ, Hricak H. CT findings of chemotherapy-induced toxicity: what radiologists need to know about the clinical and radiologic manifestations of chemotherapy toxicity. Radiology. 2011;258:41-56. 21183492 10.1148/radiol.10092129

[97] Johkoh T , Lee KS, Nishino M, et al Chest CT diagnosis and clinical management of drug-related pneumonitis in patients receiving molecular targeting agents and immune checkpoint inhibitors: a position paper from the Fleischner Society. Chest. 2021;159:1107-1125. 33450293 10.1016/j.chest.2020.11.027

[98] Dhamija E , Meena P, Ramalingam V, Sahoo R, Rastogi S, Thulkar S. Chemotherapy-induced pulmonary complications in cancer: Significance of clinicoradiological correlation. Indian J Radiol Imaging. 2020;30:20-26. 32476746 10.4103/ijri.IJRI_178_19 PMC7240883

[99] Shroff GS , Strange CD, Ahuja J, et al Imaging of immune checkpoint inhibitor immunotherapy for non-small cell lung cancer. Radiographics. 2022;42:1956-1974. 10.1148/rg.220108 36240075

[100] Albano D , Benenati M, Bruno A, et al Imaging side effects and complications of chemotherapy and radiation therapy: a pictorial review from head to toe. Insights Imaging. 2021;12:76. 34114094 10.1186/s13244-021-01017-2 PMC8192650

[101] Rotman JA , Plodkowski AJ, Hayes SA, et al Postoperative complications after thoracic surgery for lung cancer. Clin Imaging. 2015;39:735-749. 26117564 10.1016/j.clinimag.2015.05.013

[102] Tada T , Fukuda H, Matsui K, et al Non-small-cell lung cancer: reirradiation for loco-regional relapse previously treated with radiation therapy. Int J Clin Oncol. 2005;10:247-250. 10.1007/s10147-005-0501-1 16136369

[103] Hamada A , Soh J, Mitsudomi T. Salvage surgery after definitive chemoradiotherapy for patients with non-small cell lung cancer. Transl Lung Cancer Res. 2021;10:555-562. 10.21037/tlcr-20-453 33569336 PMC7867739

[104] Palma DA , Olson R, Harrow S, et al Stereotactic Ablative radiotherapy for the comprehensive treatment of oligometastatic cancers: long-term results of the SABR-COMET phase II randomized trial. J Clin Oncol. 2020;38:2830-2838. 10.1200/JCO.20.00818 32484754 PMC7460150

[105] Weickhardt AJ , Scheier B, Burke JM, et al Local ablative therapy of oligoprogressive disease prolongs disease control by tyrosine kinase inhibitors in oncogene-addicted non-small-cell lung cancer. J Thorac Oncol. 2012;7:1807-1814. 23154552 10.1097/JTO.0b013e3182745948 PMC3506112

[106] Chakrabarty N , Mahajan A. Imaging analytics using artificial intelligence in oncology: a comprehensive review. Clin Oncol (R Coll Radiol). 2024;36:498-513. 37806795 10.1016/j.clon.2023.09.013

[107] Tang A , Tam R, Cadrin-Chênevert A, et al Canadian association of radiologists white paper on artificial intelligence in radiology. Can Assoc Radiol J. 2018;69:120-135. 29655580 10.1016/j.carj.2018.02.002

[108] Bossuyt PM , Reitsma JB, Bruns DE, et al STARD 2015: an updated list of essential items for reporting diagnostic accuracy studies. Clin Chem. 2015;61:1446-1452. 26510957 10.1373/clinchem.2015.246280

[109] Collins GS , Reitsma JB, Altman DG, Moons KG. Transparent reporting of a multivariable prediction model for individual prognosis or diagnosis (TRIPOD): the TRIPOD statement. J Brit Surg. 2015;102:148-158. 10.1002/bjs.9736 25627261

[110] McCague C , Ramlee S, Reinius M, et al Introduction to radiomics for a clinical audience. Clin Radiol. 2023;78:83-98. 36639175 10.1016/j.crad.2022.08.149

[111] Hart GR , Roffman DA, Decker R, Deng J. A multi-parameterized artificial neural network for lung cancer risk prediction. PLoS One. 2018;13:e0205264. 30356283 10.1371/journal.pone.0205264 PMC6200229

[112] Ardila D , Kiraly AP, Bharadwaj S, et al End-to-end lung cancer screening with three-dimensional deep learning on low-dose chest computed tomography. Nat Med. 2019;25:954-961. 31110349 10.1038/s41591-019-0447-x

[113] Mahajan A , Kania V, Patil VM, et al Deep learning-based predictive imaging biomarker model for EGFR mutation status in non-small cell lung cancer from CT imaging. JCO. 2020;38:3106. 10.3390/cancers16061130 PMC10968632 38539465

[114] Song L , Zhu Z, Mao L, et al Clinical, conventional CT and radiomic feature-based machine learning models for predicting ALK rearrangement status in lung adenocarcinoma patients. Front Oncol. 2020;10:369. 10.3389/fonc.2020.00369 32266148 PMC7099003

[115] Wang C , Ma J, Shao J, et al Predicting EGFR and PD-L1 status in NSCLC patients using multitask AI system based on CT images. Front Immunol. 2022;13:813072. 35250988 10.3389/fimmu.2022.813072 PMC8895233

[116] Tian P , He B, Mu W, et al Assessing PD-L1 expression in non-small cell lung cancer and predicting responses to immune checkpoint inhibitors using deep learning on computed tomography images. Theranostics. 2021;11:2098-2107. 33500713 10.7150/thno.48027 PMC7797686

[117] Cheng G , Zhang F, Xing Y, et al Artificial intelligence-assisted score analysis for predicting the expression of the immunotherapy biomarker PD-L1 in lung cancer. Front Immunol. 2022;13:893198. 35844508 10.3389/fimmu.2022.893198 PMC9286729

[118] Gong J , Bao X, Wang T, et al A short-term follow-up CT based radiomics approach to predict response to immunotherapy in advanced non-small-cell lung cancer. Oncoimmunology. 2022;11:2028962. 35096486 10.1080/2162402X.2022.2028962 PMC8794258

[119] Barabino E , Rossi G, Pamparino S, et al Exploring response to immunotherapy in non-small cell lung cancer using delta-radiomics. Cancers. 2022;14:350. 35053513 10.3390/cancers14020350 PMC8773717

[120] Hou KY , Chen JR, Wang YC, et al Radiomics-based deep learning prediction of overall survival in non-small-cell lung cancer using contrast-enhanced computed tomography. Cancers (Basel). 2022;14:3798. 35954461 10.3390/cancers14153798 PMC9367244

[121] Chen N , Li R, Jiang M, et al Progression-free survival prediction in small cell lung cancer based on radiomics analysis of contrast-enhanced CT. Front Med (Lausanne). 2022;9:833283. 35280863 10.3389/fmed.2022.833283 PMC8911879

