# Supplementary material for: Imaging patterns and recommendations for diagnosis, staging, and management of lung cancer
Source: BJR Open. 2025 May 21;7(1):tzaf013. doi: 10.1093/bjro/tzaf013 (PMC12145177; doi:10.1093/bjro/tzaf013)
Supplement: tzaf013_Supplementary_Data [file tzaf013_supplementary_data.zip › Supplementary files 20.04.25.docx]

**Supplementary table 1 (S1): International guidelines for surveillance on imaging after definitive therapy (curative intent) for lung cancer.**

| **International recommending body** | **Surveillance guidelines** |
| --- | --- |
| National Comprehensive Cancer Network (NCCN) | **For Non-small cell lung cancer (NSCLC)**  **Stage I-II patients treated with surgery and/or chemotherapy:** CECT thorax every 6 months for 2-3 years then LDCT annually.  **Stage I-II patients treated with RT or stage III or stage IV (oligometastatic with all sites treated with definitive intent):** CECT thorax every 3-6 months for 3 years then CECT thorax every 6 months for 2 years followed by LDCT annually.  **For Small cell lung cancer (SCLC)**  CECT thorax with abdomen and pelvis every 2-6 months (more frequently in years 1–2 and less frequently thereafter)  CEMRI brain (if not available then CECT brain) every  3–4 months in the first year, then  every 6 months in the second year  and after 2 years, as clinically  indicated. |
| American Society of Clinical Oncology (ASCO) | **For NSCLC:** CECT thorax every 6 months for 2-3 years then annually.  **Additionally for SCLC:** CEMRI brain every 3 months for the first year and every 6 months for the second year in patients with stage I-III. |
| European Society of Medical Oncology (ESMO) | **For NSCLC:** CECT thorax and abdomen at least at 12 and 24 months and thereafter an CECT thorax with upper abdomen annually.  **For SCLC:** CT scans every 3-6 months for 2 years and increased interval thereafter.  MRI brain every 3 months in the first year and then every 6 months thereafter in those who did not receive prophylactic cranial irradiation (PCI) |

**Supplementary table 2 (S2): Indications and contraindications of lung ablative procedures.**

| **Indications** | **Contraindications** |
| --- | --- |
| *Primary lung cancer:* Inoperable (cardio-respiratory comorbidity or insufficient vital lung reserve) NSCLC stage IA  *Metastases:* Up to 3 lung nodules with contraindications to surgery. | a. SCLC.  b. Stage II NSCLC  c. Presence of nodal and distant metastases.  d. Untreatable coagulopathies.  e. Eastern Cooperative Oncology Group (ECOG) >2 and life expectancy of less than 1 year.  f. End-stage lung disease and/or respiratory failure. |

**Supplementary material 3 (S3)**

**Synoptic reporting template 1: Pre-treatment Lung Cancer Imaging - Reporting and Data System (LCI-RADS) based on CECT or FDG-PET/CT**

Demographics (Information provided by RIS and DICOM headers)

Name of the facility where examination was provided

a. Name of the patient

b. Patient’s gender

c. Patient’s date of birth and age

d. Name(s) of referring physician(s) or other health care provider(s)

e. Name of type of examination

f. Date and time of the examination

g. Date and time of dictation and final transcription

Relevant clinical information

a. Clinical symptoms

b. Addictions – Smoking/Alcohol/ Chewing tobacco

c. Co-existing health morbidities –COPD/ Diabetes Mellitus/ Immunocompromised status

d. Occupational history for any relevant occupational exposures

e. Previous history of cancer

f. Previous surgery

g. Previous chemotherapy or radiation

h. Current working diagnoses (if any)

i. Recent most relevant lab tests and/or imaging tests

j. Molecular pathology results: mutations (EGFR, ALK, BRAF, KRAS, RET, NTRK,

PDL1, ROS1, MET)

Body of the report:

a. Type of study and the technical protocol

b. Contrast information

c. Quality of examination

**Findings:**

Acute findings

*Pneumothorax:* Present/ Absent

*Pulmonary thromboembolism:* Present/ Absent

**T stage**

Lung Mass^*#^

*Location:*

*Size:*

*Shape and margins:* Spiculated / Round / Lobulated/ Smooth

*Consistency:* Centrally calcified/ peripherally calcified/ ground glass opacity / fatty attenuation/solid without any calcification

SUV max (FDG-PET/CT):

Local extent (Direct extension from lung mass)

*Airway involvement:* Absent/Main bronchus/ Carina/ Trachea

*Pleura:* Involved/ Not involved

*Chest wall:* Involved/ Not involved

*Pericardium^#^:* Involved/ Not involved

*Diaphragm:* Involved/ Not involved

*Heart^#^:* Involved/ Not involved

*Great vessels^#^:* Involved/ Not involved

*Esophagus:* Involved/ Not involved

*Spine^#^:* Involved/ Not involved

*Additional lung nodule:* Absent/ Same lobe/ Different lobe of ipsilateral lung

**N stage**

Lymph nodes^*^

*Anatomical list of suspicious nodes with their stations:*

*Size:*

*Level of suspicion:*

*Characterization, if relevant:*

SUV max of suspicious nodes (FDG-PET/CT):

**M stage**

I. Intrathoracic metastasis: Present/ Absent

*Additional nodule:* Contralateral lung/ Pleura/ Pericardium/ None

*Pleural Effusion:* Present ^$^/ Absent

*Pericardial effusion:* Present ^$^/ Absent

*Lymphangitic spread:* Present / Absent

II. Extrathoracic metastasis: Present/ Absent

*Hepatic:* Present/ Absent

*Adrenal:* Present/ Absent

*Skeletal^#^:* Present/ Absent

*Ascites:* Present/ Absent

*Brain^#^:* Present/ Absent

SUV max (FDG-PET/CT):

Associated lung parenchymal diseases

*Interstitial lung disease (ILD):* Present / Absent Extent (if present):

*Chronic obstructive pulmonary disease (COPD):* Present / Absent Extent (if present):

Other significant findings:

**Impression**

Imaging based TNM stage

* Needs biopsy correlation

# Needs additional imaging: MRI of the brachial plexus in case of pancoast tumour to rule out brachial plexus involvement/MRI thorax as a problem solving tool in case of indeterminate involvement of pericardium, great vessels, or heart on CECT/ MRI spine to look for intraspinal extension and cord compression in case of vertebral involvement/ MRI for detection of bone marrow disease in SCLC/MRI brain for better evaluation of brain metastasis

$ Fine needle aspiration with cytology testing needed to confirm malignant cells in effusion

CECT: Contrast Enhanced Computed Tomography, FDG-PET/CT: Fluorodeoxyglucose Positron Emission Tomography Computed Tomography , RIS: Radiology Information System,

DICOM: Digital Imaging and Communications in Medicine, COPD: Chronic Obstructive Pulmonary Disease, Epidermal growth factor receptor (EGFR), Anaplastic lymphoma kinase (ALK), BRAF (v-Raf murine sarcoma viral oncogene homolog B), Neurotrophic tyrosine receptor kinase (NTRK), ROS proto-oncogene 1 (ROS1), KRAS (Kirsten rat sarcoma virus), mesenchymal-epithelial transition (MET), RET (rearranged during transfection), programmed death ligand-1 (PD-L1)

SUV max: Maximum Standardized Uptake Value, TNM: Tumour Node Metastasis, MRI: Magnetic Resonance Imaging, SCLC: Small cell lung cancer

**Supplementary material 4 (S4)**

**Synoptic reporting template 2: Post-therapy Lung Cancer Imaging - Reporting and Data System (pLCI-RADS) based on CECT or FDG-PET/CT**

Demographics (information provided by RIS and DICOM headers)

Name of the facility where examination was provided

a. Name of the patient

b. Patient’s gender

c. Patient’s date of birth and age

d. Name(s) of referring physician(s) or other health care provider(s)

e. Name of type of examination

f. Date and time of the examination

g. Date and time of dictation and final transcription

Relevant clinical information

a. Clinical symptoms

d. Recent most relevant lab tests and/or imaging findings

**Indication:** [Post surgery/ post NACT/ post chemotherapy/ post radiotherapy/ post immunotherapy/ post combined systemic therapy]

Body of the report:

a. Type of study and the technical protocol

b. Quality of examination

c. Comparison to previous study and date

d. Time (in weeks) after treatment at the time of scan

**Findings:**
Acute findings

*Pneumothorax:* Present/ Absent

*Pulmonary thromboembolism:* Present/ Absent

1. Residual disease/recurrence at the primary site: Present/ Absent

Location (if present):

Size (if present):

SUV _peak_:

2. Residual pathological lymph nodes /new pathological lymph nodes: Present/ Absent

Location (if present):

Size (if present):

SUV _peak_:

3. Indeterminate, requires FNA/biopsy correlation or follow up imaging after at least 4 weeks (to account for pseudoprogreesion after immunotherapy).

4. Any aggressive lesions suspicious for metastatic involvement in visualized portions of liver, adrenals, and bones: Present/ Absent

5. Post treatment lung changes / injury:

A. Hyperprogression on immunotherapy

B. Acute changes: Diffuse alveolar damage/Hypersensitivity pneumonitis/Organizing pneumonia/Sarcoidosis-like pattern/ Radiation pneumonitis
C. Late changes: Fibrotic Non-specific interstitial pneumonia /Radiation recall pneumonitis

**IMPRESSION:**
1. Mention type of post treatment lung changes/injury (if present)

2. Residual/Recurrent disease post-surgery (if present)

3. Mention post therapy response as per RECIST 1.1/PERCIST/iRECIST *

***CECT or FDG-PET/CT Surveillance Legend**

*Respo*nse *on CECT* *as per RECIST 1.1*

1. Complete response

2. Partial response

3. Progressive disease

4. Stable disease

*Respo*nse *on CECT* as per iRECIST (post immunotherapy)

1. Immune related Complete response (iCR)

2. Immune related Partial response (iPR)

3. Immune related unconfirmed progressive disease (iUPD)

4. Immune related confirmed progressive disease (iCPD)

4. Immune related Stable disease (iSD)

*Respo*nse *on FDG-PET/CT* *as per PERCIST 1.0*

1. Complete metabolic response

2. Partial metabolic response

3. Progressive metabolic disease

4. Stable metabolic disease

**Recommendation**

None

Routine follow-up

Close follow-up

CECT: Contrast Enhanced Computed Tomography, FDG-PET/CT: Fluorodeoxyglucose Positron Emission Tomography Computed Tomography , RIS: Radiology Information System, DICOM: Digital Imaging and Communications in Medicine, NACT: Neoadjuvant Chemotherapy , SUL _peak_: Standardized uptake value corrected for lean body mass, FNA: Fine Needle Aspiration, RECIST: Response Evaluation Criteria in Solid Tumors, PERCIST: Positron Emission Tomography Response Criteria In Solid Tumors, iRECIST: Immune Response Evaluation Criteria In Solid Tumor
